# Small Modular Electrochemical Containers for Decarbonization of AI Data Centers

**DOI:** 10.1021/acs.est.6c06512

**Published:** 2026-10-29

**Authors:** Ahmad Hassan, Prince Aleta, Mohsen Afshari, Abdelrahman Refaie, Mim Rahimi

**Affiliations:** † Department of Civil and Environmental Engineering, 14743 University of Houston, Houston, Texas 77204, United States; ‡ Materials Science and Engineering Program, University of Houston, Houston, Texas 77204, United States

**Keywords:** AI Energy Infrastructure, Data Center, Decarbonization, Carbon Capture, Electrochemistry, EMAR

## Abstract

The rapid growth of artificial intelligence (AI) is increasing data-center electricity demand and reinforcing the near-term role of fossil-fuel power generation. Decarbonizing this infrastructure requires carbon capture systems that are efficient, compact, modular, and compatible with space-constrained data centers. Here, we evaluate Small Modular Electrochemical Containers (SMECs) as a deployment-oriented application of a two-stage, membraneless electrochemically mediated amine regeneration (EMAR) process. The design integrates CO_2_ absorption and electrochemical desorption within one gas-diffusion-electrode cell, eliminating absorber columns and ion-exchange membranes. Single-cell experiments were conducted under simulated natural-gas and coal-derived flue gas. Polarization mapping identified optimized operating conditions, which were then used in a 300-cycle, 50-h accelerated stability test with periodic polarity switching. The cell maintained repeatable voltage and CO_2_ absorption/desorption profiles, with absorption and desorption efficiencies of ∼90% under optimized conditions. The energetics ranged from 50–85 kJe/molCO_2_, and GC/IC analyses showed no detectable gaseous or liquid side products. The paper further includes theoretical modeling of SOx and NOx effects on the absorbent. In a case study for a 360 MW data-center power plant, the TEA showed that fewer than 20 SMEC units could achieve near-complete decarbonization, increasing the levelized electricity cost from about $58/MWh to $74/MWh.

## Graphical abstract

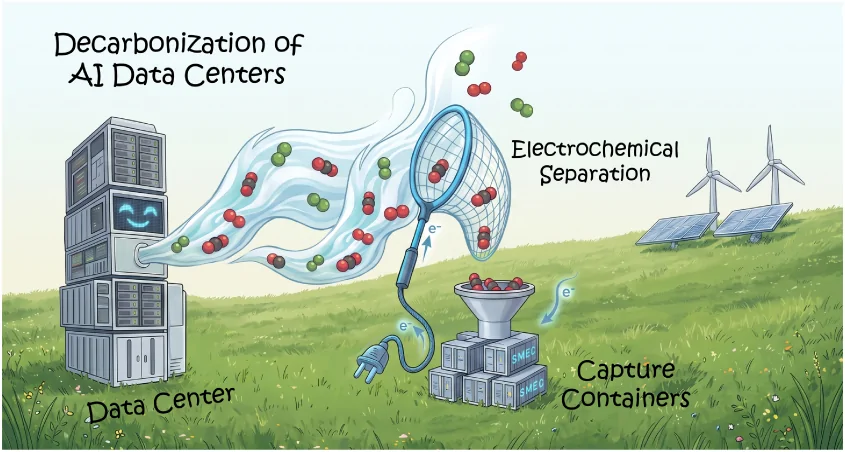

## 1 Introduction

Artificial intelligence (AI) began its explosive growth in 2022, driving a corresponding surge in data center construction.^1–4^ Data centers are specialized facilities that house servers and networking equipment to process, store, and distribute digital information. This emerging AI economy and the corresponding energy demands of data centers are fueling unprecedented growth in U.S. electricity consumption.^5–7^ According to the U.S. Department of Energy (DOE), data centers accounted for approximately 4.4% of total U.S. electricity use in 2023 and are projected to comprise 6.7–12% by 2028. The associated greenhouse gas (GHG) emissions were estimated at 61 Mt CO_2_ equivalent in 2023, which represents roughly 1% of total U.S. GHG emissions.^8^ These trends highlight the urgent need for innovative strategies to mitigate GHG emissions, especially CO_2_, from data-center power generation.

There are several low-emission options for powering data centers, including nuclear, geothermal, and renewables paired with battery storage.^6,9^ However, these technologies may not always match the cost, reliability, or scalability of fossil-fuel power plants. AI data centers demand continuous, around-the-clock operation and typically draw on the order of 100–1000 MW.^8^ While fossil-fuel plants can reliably deliver large-scale power at low cost, their associated high GHG emissions remain a significant concern.

Carbon capture and storage (CCS) has demonstrated its effectiveness in reducing GHG emissions from fossil-fuel power generation by separating CO_2_ from the generated flue gas, which can be subsequently sequestered.^10^ By integrating CCS, a power plant can continue to deliver reliable, cost-effective electricity at scale while substantially lowering its carbon footprint.^11^ Furthermore, CCS-equipped fossil-fuel power plants can be coupled with renewables to create hybrid low-emission energy portfolios for data centers.

An optimal CCS solution for AI data-center applications should meet several key criteria. First, it must be cost-effective, since any CO_2_ capture step raises the delivered price of electricity. Second, high energy efficiency is essential to minimize the fraction of generated power consumed by the capture process itself. Third, a compact footprint and straightforward installation are critical for retrofits or constrained sites. Fourth, modularity and flexible capacity scaling allow operators to match capture rates to fluctuating demand. Finally, proven maturity and long-term operational durability are necessary to ensure continuous, low-maintenance performance over the plant’s lifetime.

The most conventional CCS technology for point sources (e.g., power plants) relies on amine scrubbing with thermal regeneration. In an absorber, CO_2_ reacts selectively with an amine solvent (e.g., monoethanolamine, MEA), producing a CO_2_-rich solution that is then heated in a desorber to release pure CO_2_ and regenerate the amine for recycle. Despite its commercial maturity, this process imposes a high energy penalty and incurs high rates of solvent degradation.^12–15^ When applied to power plants serving AI data centers, further challenges arise. These systems demand large footprints and complex retrofit layouts, making integration into existing sites difficult. They also lack operational flexibility; scaling capture capacity requires major equipment modifications rather than simple add-ons.

Electrochemical carbon capture (ECC) is rapidly emerging as an alternative to conventional thermal-swing CCS.^16–21^ The fundamental distinction lies in the nature of energy input: while conventional systems rely on thermal swings to regenerate absorbents, ECC systems often employ electron swings driven by redox reactions. This electrochemical pathway inherently eliminates many limitations of conventional thermal processes. Operation at ambient temperature minimizes solvent degradation. In addition, the electricity-driven cycle achieves higher energy efficiency by avoiding large parasitic heat demands.^16,22^ ECC architectures, drawing on battery and fuel-cell design, enable compact, stackable modules that are straightforward to retrofit. Moreover, capture capacity can be flexibly scaled by simply adding or removing modules. These intrinsic advantages make ECC especially attractive for AI data center power plants, where rapid deployment, site constraints, and variable load profiles demand adaptable CCS solutions.

Depending on the redox mechanism employed, ECC systems can be broadly classified into direct and indirect approaches. In the direct route, redox-active species participate directly in CO_2_ separation and concentration. Upon electrochemical reduction, they selectively bind CO_2_ from a gas mixture (e.g., flue gas), and upon oxidation, release and concentrate it.^16,17^ The most studied direct method involves electrochemical generation of nucleophiles (EGN), with quinones representing the most extensively developed redox class.^23–28^ When reduced, quinones form nucleophilic sites that react with the electrophilic carbon of CO_2_, enabling selective capture over other gases. The reversal in polarity results in releasing and concentrating CO_2_. However, this mechanism often faces a critical limitation of oxygen sensitivity. Both chemical and electrochemical side reactions involving O_2_ can degrade the redox carrier or suppress its activity, leading to performance losses. Given the significant oxygen content in flue gases (between 3% to 15%), direct ECC routes may face practical barriers for implementation in power plants serving data centers.

In the indirect approaches, redox reactions facilitate the carbon capture process without directly binding CO_2_. These reactions may mediate the separation stage, the concentration stage, or both. Two major indirect ECC technologies have been developed: pH swing^29–35^ and electrochemically mediated amine regeneration (EMAR).^36–41^ In the pH swing process, proton-coupled electron transfer (PCET) reactions drive pH changes, raising the pH at the cathode upon reduction and lowering it at the anode upon oxidation. This electrochemically induced pH gradient, when coupled with the pH sensitivity of CO_2_ hydration, enables CO_2_ capture at high pH and release at low pH. A major limitation of this approach lies in the sluggish kinetics of CO_2_ hydration.^12,42^ This reaction-rate limitation necessitates large absorber volumes to ensure sufficient gas-liquid contact time, resulting in bulky systems that are poorly suited for space-constrained environments such as data center power infrastructure, where compactness and high capture throughput are essential. EMAR addresses this constraint by utilizing the same fast-kinetics carbamate formation in the absorber as in conventional amine scrubbing, while replacing the thermal desorber with a room-temperature electrochemical cell, where CO_2_ is released, and the amine is regenerated via a redox-mediated process. As such, EMAR offers a promising indirect ECC route for integration into fossil fuel power plants aimed at data center decarbonization.

The early developed EMAR was a four-stage process.^36,37^ In the first stage, CO_2_ was selectively separated from flue gas (which contains N_2_ and O_2_) using ethylenediamine (EDA) as the absorbent. EDA was used because the Cu-mediated EMAR cycle requires an amine that absorbs CO_2_ and coordinates Cu^2+^ strongly to form a reversible Cu­(EDA)_2_^2+^ complex. The CO_2_-rich stream with EDA-CO_2_ was introduced to the anode chamber of a two-compartment electrochemical cell, where oxidation of the copper electrode (Cu^0^ → Cu^2+^ + 2e^–^) results in complexation of copper ions with EDA and subsequent liberation of CO_2_ (Cu^2+^ + 2EDA-CO_2_ → Cu­(EDA)_2_^2+^ + 2CO_2_) (stage 2). After removal of the desorbed CO_2_ in a flash tank (stage 3), the CO_2_-lean stream is sent to the cathode chamber, where electrodeposition of copper from the complex results in reactivating the EDA absorbent (Cu­(EDA)_2_^2+^ + 2e^–^ → Cu^0^ + 2EDA) (stage 4). The outlet of the cathode chamber is sent back to the absorber to close the loop. Further details of the four-stage EMAR are provided in Section S1.1 of the *Supplementary Information*.

Various four-stage EMAR processes incorporating different electrodes, absorbents, and additives have been developed for CO_2_ capture from flue gas. Both fundamental and applied studies have advanced the understanding of this system (refer to Section S1.2 of the *Supplementary Information*). The process demonstrated energy requirements that are competitive with those of conventional thermal carbon capture technologies. Reported energy consumptions for EMAR systems have ranged from 35 to 150 kJ/molCO_2_, placing them within the range of conventional MEA-based processes but with the added advantage of operating at ambient temperature. Unlike most ECC approaches, which remain at lab-scale proof-of-concept stages, EMAR has been scaled up to sub-pilot level and operated in an automated, continuous mode for over 100 h using a simulated flue gas.^37,40^ The system exhibited a stable capture profile approaching the theoretical capacity and showed strong resilience to high concentrations of oxygen in the gas stream.

Although the four-stage EMAR system is among the most advanced ECC technologies, its implementation for decarbonizing data centers still presents two main challenges. First, while the electrochemical desorber unit is inherently modular—allowing capacity to be increased by stacking additional cells—the process as a whole lacks modularity due to the absorber column. Gas-liquid absorption units are typically large, custom-engineered systems with limited scalability by duplication, making them unsuitable for fine-grained capacity adjustments. As a result, the flexibility to tune capture capacity by simply adding or removing electrochemical stacks is lost. Second, the use of ion-selective membranes to separate the two chambers of the electrochemical cell introduces significant technical and economic burdens. A recent technoeconomic analysis (TEA) estimated that membranes account for approximately 50% of the total capital cost,^43^ constituting the primary cost driver behind the reported capture cost of ∼$140 per tonne of CO_2_. In addition to the high cost, membrane fouling has been often observed,^44^ necessitating periodic disassembly and replacement, which contributes to elevated operational expenditures.

To eliminate these challenges, we recently introduced the concept of a two-stage EMAR system by fundamentally redesigning the process architecture.^45^ A compact electrochemical cell incorporating two gas diffusion electrodes (GDEs) was developed to integrate both absorption and desorption within a single enclosure. Each GDE consists of a gas diffusion layer and a mesoporous scaffold for enhanced gas-liquid contact, along with copper electrodeposited to facilitate the redox reactions (Figure 1A). In stage 1, EDA is regenerated locally at the cathode GDE, enabling selective CO_2_ capture from the adjacent gas channel. The resulting EDA-CO_2_ complex diffuses to the anode GDE, where copper oxidation drives complexation to form Cu­(EDA)_2_^2+^ and release CO_2_, which exits through the anode gas channel (stage 2). The generated Cu­(EDA)_2_^2+^ complex then returns to the cathode GDE via diffusion and migration, completing the closed-loop cycle (Figure 1B). This new two-stage EMAR concept eliminates the need for both the absorption column and the ion-selective membrane, enabling a compact, integrated system ideally suited for data center decarbonization. In this membraneless architecture, transport between electrodes is not treated as an undesired crossover, rather it is required by the system to work efficiently. Our TEA estimated CO_2_ capture costs as low as $50 per tonne,^45^ further highlighting the cost competitiveness of this compact electrochemical approach.

**Figure 1:**
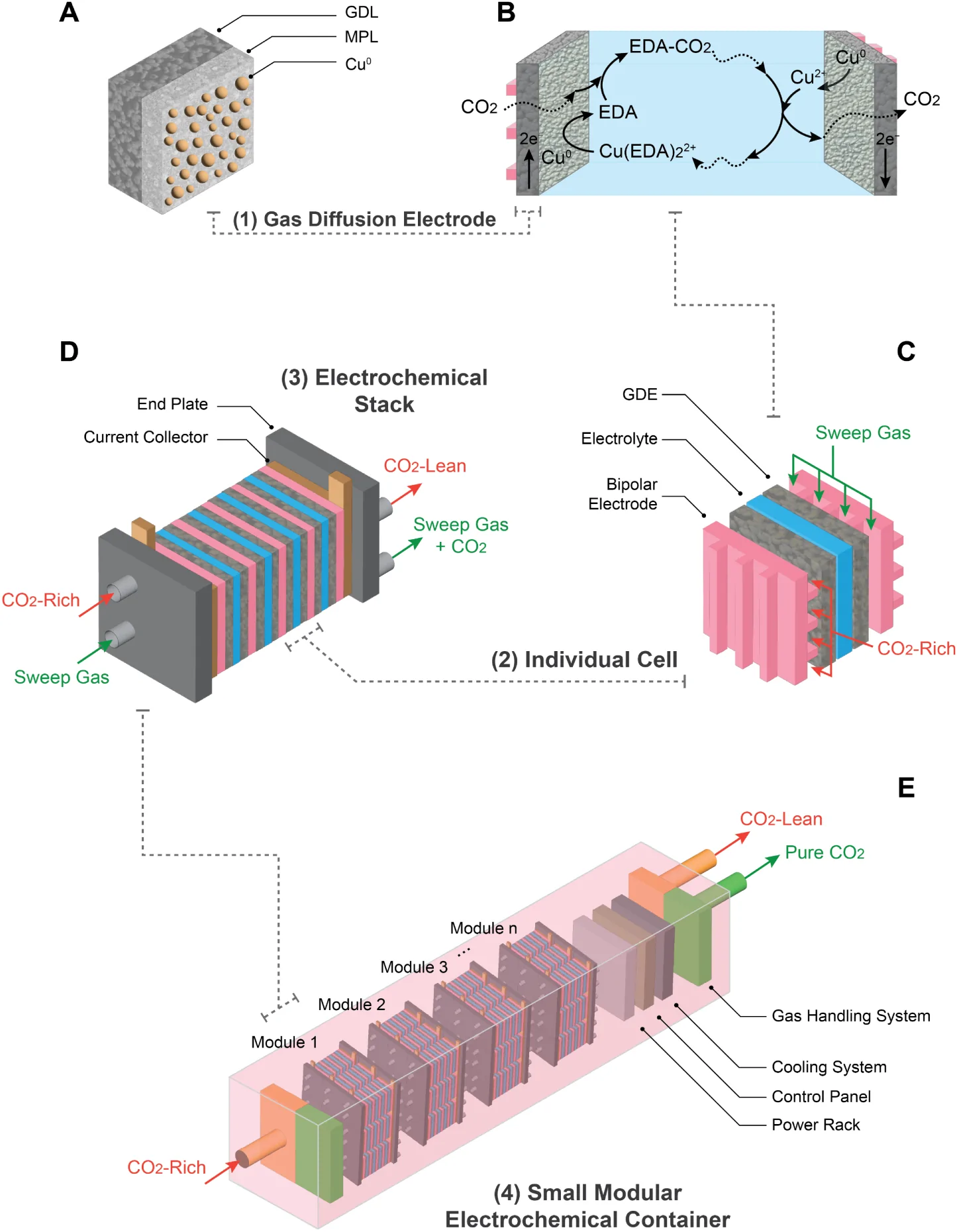
Architecture and scaling pathway of the two-stage EMAR system toward SMECs. (A) Schematic of the gas diffusion electrode (GDE) used in the two-stage EMAR system, consisting of a gas diffusion layer (GDL), a mesoporous layer (MPL) to enhance gas-liquid interfacial contact, and electrodeposited copper (Cu^0^) particles that serve as the redox-active phase. (B) Operating principle of the two-stage membraneless EMAR process within a single electrochemical cell. At the cathode GDE, electrodeposition of copper from the Cu­(EDA)_2_^2+^ complex regenerates free ethylenediamine (EDA), enabling localized CO_2_ absorption from the adjacent gas channel to form EDA-CO_2_. The carbamate complex diffuses through the electrolyte toward the anode GDE, where copper oxidation (Cu^0^→ Cu^2+^+ 2e^–^) drives complexation with EDA and releases CO_2_ into the anode gas channel. The Cu­(EDA)_2_^2+^ complex then returns to the cathode via migration and diffusion, closing the electrochemical loop without the use of an ion-selective membrane. (C) Schematic of an individual electrochemical cell, highlighting the bipolar electrode architecture, electrolyte compartment, and two GDEs, and two gas channels for CO_2_-rich inlet gas and sweep gas. (D) Assembly of multiple individual cells into an electrochemical stack, including current collectors and end plates, illustrating the scalable stacking strategy that enables increased CO_2_ capture capacity through modular duplication. (E) Conceptual design of a Small Modular Electrochemical Container (SMEC), in which multiple electrochemical modules are housed within a standard 40-foot shipping container and integrated with balance-of-plant components, including gas-handling systems, cooling infrastructure, power electronics, and control hardware. CO_2_-rich flue gas enters the container, while CO_2_-lean gas and CO_2_-enriched streams exit separately, enabling plug-and-play deployment for megawatt-scale carbon capture applications.

While our previous work established the architectural concept of the two-stage membraneless EMAR process, it did not determine whether that architecture could operate as a practical carbon-capture system under continuous flue gas supply conditions. The present study evaluates this architecture as a deployment-oriented single-cell platform for fossil-fuel power plants supplying AI data centers. Specifically, the process is evaluated under realistic flue gas compositions representative of natural gas (5% CO_2_) and coal combustion (13% CO_2_), and full polarization mapping is used to define the operating window and optimal current regime for each case. A 300-cycle, 50-h accelerated polarization-switching test was then used to assess the operational stability of the cell under these optimized conditions. The effects of SO_2_ and NO were evaluated through combined thermodynamic and electrochemical modeling, which provides an impurity-risk assessment rather than an experimental impurity-tolerance demonstration. The stack, module, and container drawings in Figures 1 and 2 are therefore used as a scaling pathway and TEA framework, not as experimental validation of a complete containerized SMEC unit.

**Figure 2:**
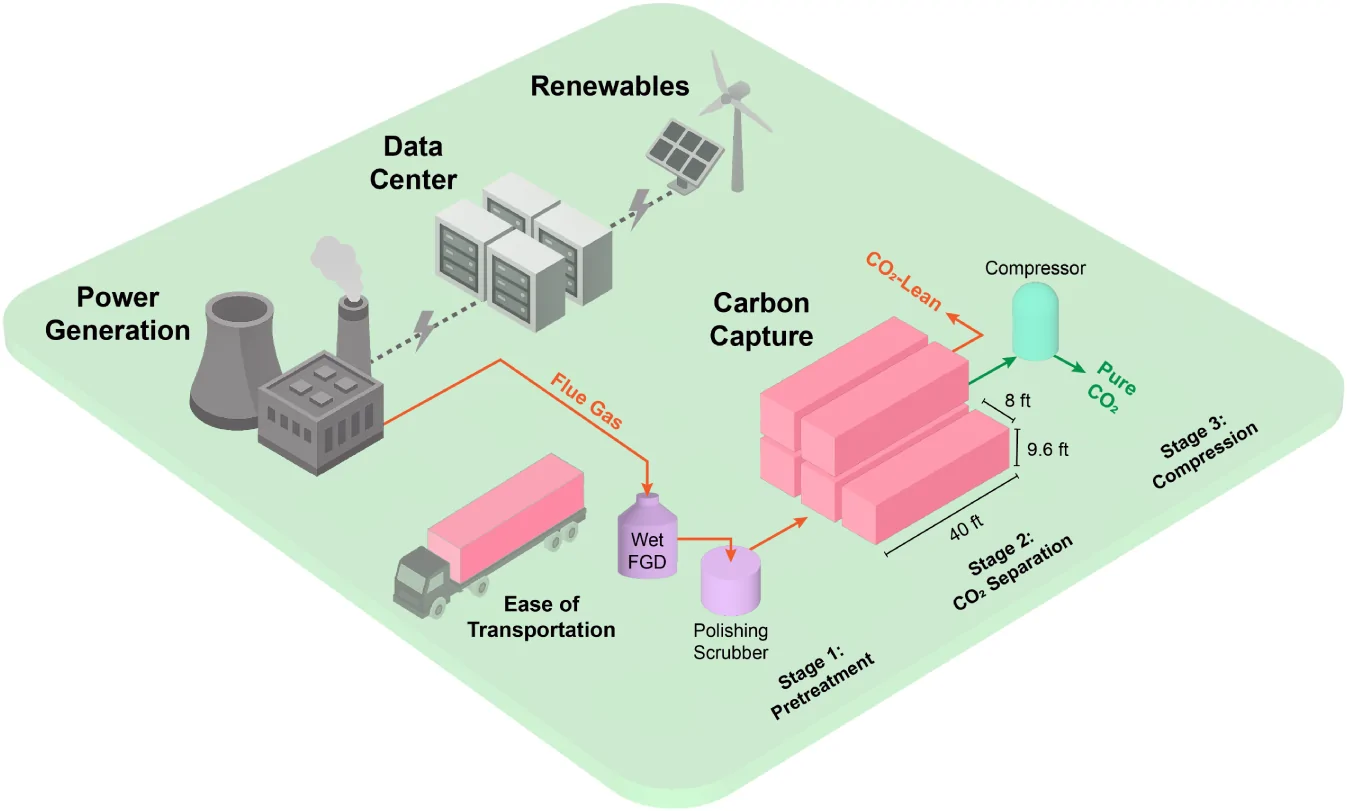
System-level integration of SMECs for data-center decarbonization. Schematic illustration of the deployment of SMEC technology for carbon capture from a fossil-fuel-based power plant supplying an AI data center. Electricity is generated on-site using a fossil fuel-based power plant and supplemented by renewable energy sources (wind and solar) to meet the data center’s electrical demand. CO_2_-containing flue gas from the power plant is routed to a compact pretreatment train (if required), consisting of wet flue gas desulfurization (FGD) followed by a polishing scrubber to remove trace acid-gas impurities. The conditioned flue gas is then directed to the SMEC-based carbon capture system, where CO_2_ is electrochemically separated within containerized modules housed in standard 40-foot shipping containers. The SMECs produce a CO_2_-lean exhaust stream and a CO_2_-enriched stream in the anode outlet. Further downstream concentration and compression would be required to generate a storage-ready CO_2_ product. The modular, containerized architecture enables ease of transportation, parallel deployment, and scalable integration with megawatt-scale power generation infrastructure, making the system well-suited for data-center decarbonization applications.

Building on this development platform, we established a scaling pathway from single electrochemical cells (Figure 1C) to stacked assemblies (Figure 1D), modular units, and ultimately to Small Modular Electrochemical Containers (SMECs) (Figure 1E). Each SMEC adopts a standard 40-foot shipping container form factor and houses multiple modules together with integrated power electronics, cooling, and process controls. SMECs can be transported, deployed, and operated in parallel to achieve plug-and-play CO_2_ capture from a megawatt-scale power plant (Figure 2). To evaluate deployment feasibility under a realistic AI-driven industrial demand, we further performed a case study of full-scale SMEC implementation for OpenAI’s 360 MW Stargate data center currently under construction in Abilene, Texas. This analysis quantified the number of SMEC units required, the electricity cost impact, and the pretreatment requirements associated with natural gas-derived flue gas, providing a technoeconomic and systems-level perspective on SMEC integration for AI data center decarbonization.

Schematic illustration of the deployment of SMEC technology for carbon capture from a fossil-fuel-based power plant supplying an AI data center. Electricity is generated on-site using a fossil fuel-based power plant and supplemented by renewable energy sources (wind and solar) to meet the data center’s electrical demand. CO_2_-containing flue gas from the power plant is routed to a compact pretreatment train (if required), consisting of wet flue gas desulfurization (FGD) followed by a polishing scrubber to remove trace acid-gas impurities. The conditioned flue gas is then directed to the SMEC-based carbon capture system, where CO_2_ is electrochemically separated within containerized modules housed in standard 40-foot shipping containers. The SMECs produce a CO_2_-lean exhaust stream and a CO_2_-enriched stream in the anode outlet. Further downstream concentration and compression would be required to generate a storage-ready CO_2_ product. The modular, containerized architecture enables ease of transportation, parallel deployment, and scalable integration with megawatt-scale power generation infrastructure, making the system well-suited for data-center decarbonization applications.

## 2 RESULTS & DISCUSSION

### 2.1 System-Level Demonstration

The system-level validation of the two-stage membraneless EMAR process was conducted under two representative flue gas compositions corresponding to major AI data center deployment pathways. A mixture containing 5% CO_2_, 13% O_2_, and 82% N_2_ was used to simulate the flue gas from a natural gas-fired power plant, whereas a mixture of 13% CO_2_, 4% O_2_, and 83% N_2_ represented typical coal-derived flue gas. Prior to system operation, the redox behavior of the Cu-EDA-CO_2_ cycle under both gas compositions was characterized through cyclic voltammetry (CV) and electrochemical impedance spectroscopy (EIS) to resolve the electron-transfer thermodynamics, interfacial kinetics, and transport resistances. These electrochemical characterizations were essential for understanding the active species behavior under implementation-relevant conditions and are reported in Section S2 of the Supplementary Information. The system development continued with polarization analysis, used to identify the optimal operating current. This was followed by long-term continuous operation under the selected condition to assess process durability and operational stability under realistic flue gas exposure.

#### 2.1.1 Polarization Behavior and Optimal Operating Condition

The primary objective of the polarization experiment was to determine the optimal operating current for CO_2_ capture from natural gas- and coal-derived flue gas compositions. At each applied current, two key outputs were measured: the cell voltage and the CO_2_ uptake at the cathode GDE and the CO_2_ release at the anode GDE. Based on preliminary screening experiments, a current range of 25–75 mA (23–69 A/m^2^) was selected for the natural gas scenario, while a broader range of 25–125 mA (23–116 A/m^2^) was investigated for the coal scenario to account for its higher CO_2_ loading. These current-density values define the practical operating window for the present GDE design. The highest applied currents did not provide the best performance because gas transport began to limit the electrochemical regeneration rate. Scale-up should therefore rely on increasing active area through improved GDE structure, optimized gas-channel distribution, and reduced ohmic losses from electrolyte resistance, rather than only increasing the current in a single cell. These measurements were then used to calculate three performance metrics, including (i) energetics (kJ electricity per mol CO_2_ separated; kJ_e_/molCO_2_), (ii) single-pass CO_2_ capture efficiency (SPE), and (iii) Faradaic efficiency (FE). The SPE was defined as the percentage of CO_2_ uptake at the cathode relative to the total inlet CO_2_ supplied during the same absorption period; however, FE represents the fraction of charge directed toward the Cu-EDA redox cycle. For clarity, SPE is used as the absorption efficiency because it represents cathodic CO_2_ absorption during negative polarization, while FE is used as the desorption efficiency because it quantifies charge-driven CO_2_ release during anodic operation.

As expected for a kinetically mediated redox process, the cell voltage increased with applied current for both natural gas and coal scenarios, consistent with Butler–Volmer kinetics,^39^ where higher overpotential is required to sustain larger reaction rates at the electrode interface. In parallel, both absolute CO_2_ absorption (at the cathode) and desorption (at the anode) increased with current, reflecting the stronger electrochemical driving force generated under higher electron-transfer rates (Figure 3A, 3B). However, analysis of energy requirement revealed that neither extreme of the polarization range delivered optimal performance. At the lowest applied current (25 mA for both natural gas and coal scenarios), energy consumption was high due to insufficient driving force for CO_2_ release, i.e., a small amount of CO_2_ was desorbed relative to input power, resulting in poor power utilization. Conversely, at the highest applied current (75 mA for natural gas and 125 mA for coal), the energetic penalty again rose, but this time due to a mass-transport limitation: although electrical power input increased, CO_2_ absorption could not keep pace with the regeneration of amine, leading to diminishing marginal gains in desorbed CO_2_ (Figure 3C, 3D). Thus, high current did not translate to proportionally higher CO_2_ throughput, making the process energetically unfavorable at that extreme as well. These two limiting regimes were generally consistent with the behavior of desorption efficiency, defined as the fraction of input current utilized toward CO_2_ release. Efficiency dropped significantly at the lowest (kinetic underdrive) and highest applied current (mass-transfer saturation), while maintaining its highest values at intermediate currents, where kinetic and transport resistances are jointly minimized. In contrast, absorption efficiency at the cathode GDE increased steadily with applied current across the entire range (Figure 3C, 3D). This trend reflects that the local regeneration rate of free EDA, the rate-limiting precursor for carbamate formation, scales directly with electron flow. Stronger regeneration leads to higher near-surface CO_2_ capture capacity, enabling continuously increasing absorption rates with increasing current for both natural gas and coal gas compositions.

**Figure 3:**
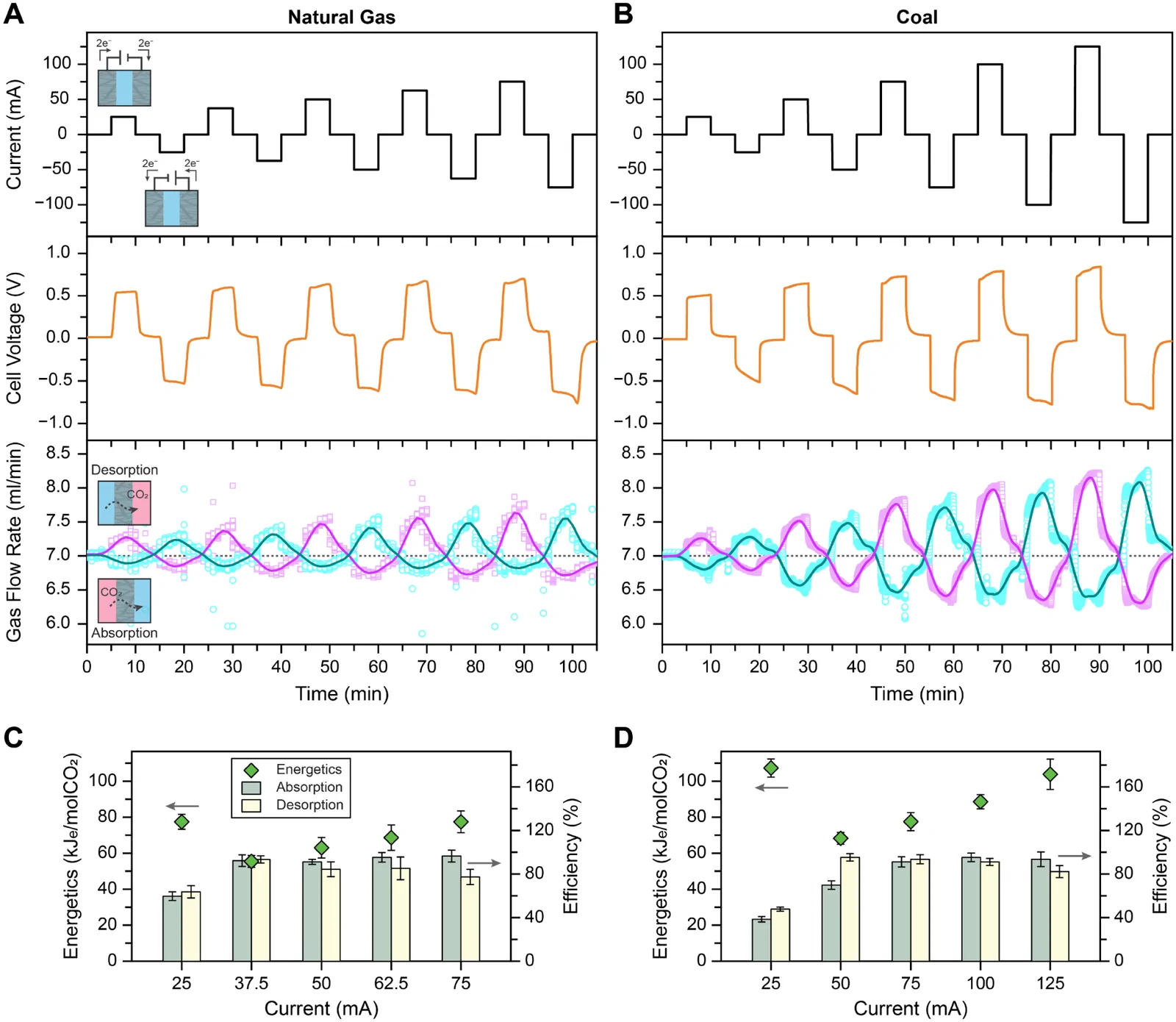
Polarization behavior and identification of optimal operating current. (A, B) Time-resolved response under periodic polarity switching showing the applied current profile (top), corresponding cell voltage (middle), and measured gas-phase CO_2_ flow rates associated with absorption at the cathode GDE and desorption at the anode GDE (bottom) for (A) natural gas, and (B) coal. Insets schematically illustrate the alternating two-electron Cu redox reactions occurring at each electrode during polarity reversal. (C, D) Calculated performance metrics as a function of applied current, including electrical energetics (kJ_e_/molCO_2_), CO_2_ absorptionefficiency representing the single-pass CO_2_ capture efficiency (SPE) at the cathode, and CO_2_ desorptionrepresenting the Faradaic efficiency (FE) at the anode for (A) natural gas, and (B) coal. Error bars represent the variance in energetics and efficiencies calculated from the steady-state portions of each polarity cycle, obtained by averaging data over the final 3 min of each polarity.

To determine the optimal operating condition, energy consumption and both desorption and absorption efficiencies were evaluated across all applied current densities. At the optimum point, the process should exhibit (i) minimum energy consumption, (ii) maximum desorption and absorption efficiencies, and (iii) minimal mismatch between absorption and desorption efficiencies, which is critical for maintaining cycle-to-cycle balance during periodic polarity switching. Since desorption efficiency (FE) for EMAR remains high under the tested conditions, comparing absorption and desorption efficiencies provides a practical check of cycle balance. A ratio close to unity (minimal mismatch) indicates that the CO_2_ absorbed at the cathode is released at the anode at a comparable rate, which is necessary for stable polarity switching. For the natural gas flue gas scenario, the lowest energy requirement was observed at 37.5 mA (57±3 kJ_e_/molCO_2_), followed by 50 mA (63±5 kJ_e_/molCO_2_). While absorption efficiencies were identical (94±3%) at both conditions, desorption efficiency was higher at 37.5 mA (95±2%) compared to 86±5% at 50 mA, resulting in near-zero mismatch between absorption and desorption efficiencies at 37.5 mA versus ∼8% at 50 mA (Figure 3C). Considering minimum energy consumption, high efficiencies, and perfect absorption/desorption balance, 37.5 mA was selected as the optimal operating current for natural gas flue gas. For the coal flue gas scenario, the lowest energy requirement occurred at 50 mA (69±2 kJ_e_/molCO_2_), followed by 75 mA (78±3 kJ_e_/molCO_2_). However, absorption efficiency at 50 mA was substantially lower (71±2%) than at 75 mA (91±3%), despite similar desorption efficiencies (95±2% vs. 94±2%). Hence, the absorption-desorption mismatch was much larger at 50 mA (∼24%) relative to 75 mA (only a few percent) (Figure 3D), indicating a higher risk of imbalance under polarity cycling. Accordingly, 75 mA was selected as the optimal operating condition for coal flue gas for subsequent long-term continuous operation.

#### 2.1.2 Accelerated Polarity-Switching Operation and Stability

The experiment was conducted for 300 electrochemical cycles over 50 h and is therefore referred to as an accelerated polarity-switching test rather than a direct demonstration of months-long field operation. Each cycle consisted of 5 min at open circuit followed by 5 min of electrochemical driving at constant current, with the cell polarity alternated every cycle. This configuration ensures that each electrode alternately functions as a cathode and an anode to avoid a complete depletion or surface blockage of the electrodes. To reflect the optimized conditions identified from polarization analysis, constant-current operation was employed at 37.5 mA (35 A/m^2^) for natural gas and 75 mA (69 A/m^2^) for coal flue gas compositions.

Reversible electrochemical cycling was maintained throughout the 300-cycle test for both natural gas and coal flue-gas compositions, as evidenced by the stable cell-voltage waveforms and repeatable CO_2_ absorption/desorption profiles (Figure 4A, 4B). Consistent with the Butler–Volmer behavior and the polarization measurements, the natural gas case operated at lower cell voltages than the coal case. The magnitude of cyclic CO_2_ uptake and release was also smaller for natural gas, which is expected, given both the lower applied current and the reduced inlet CO_2_ concentration. Despite these differences in absolute CO_2_ throughput, the cycle-averaged absorption and desorption efficiencies remained high (near 90% in both scenarios), mirroring the performance trends observed in the polarization evaluation.

**Figure 4:**
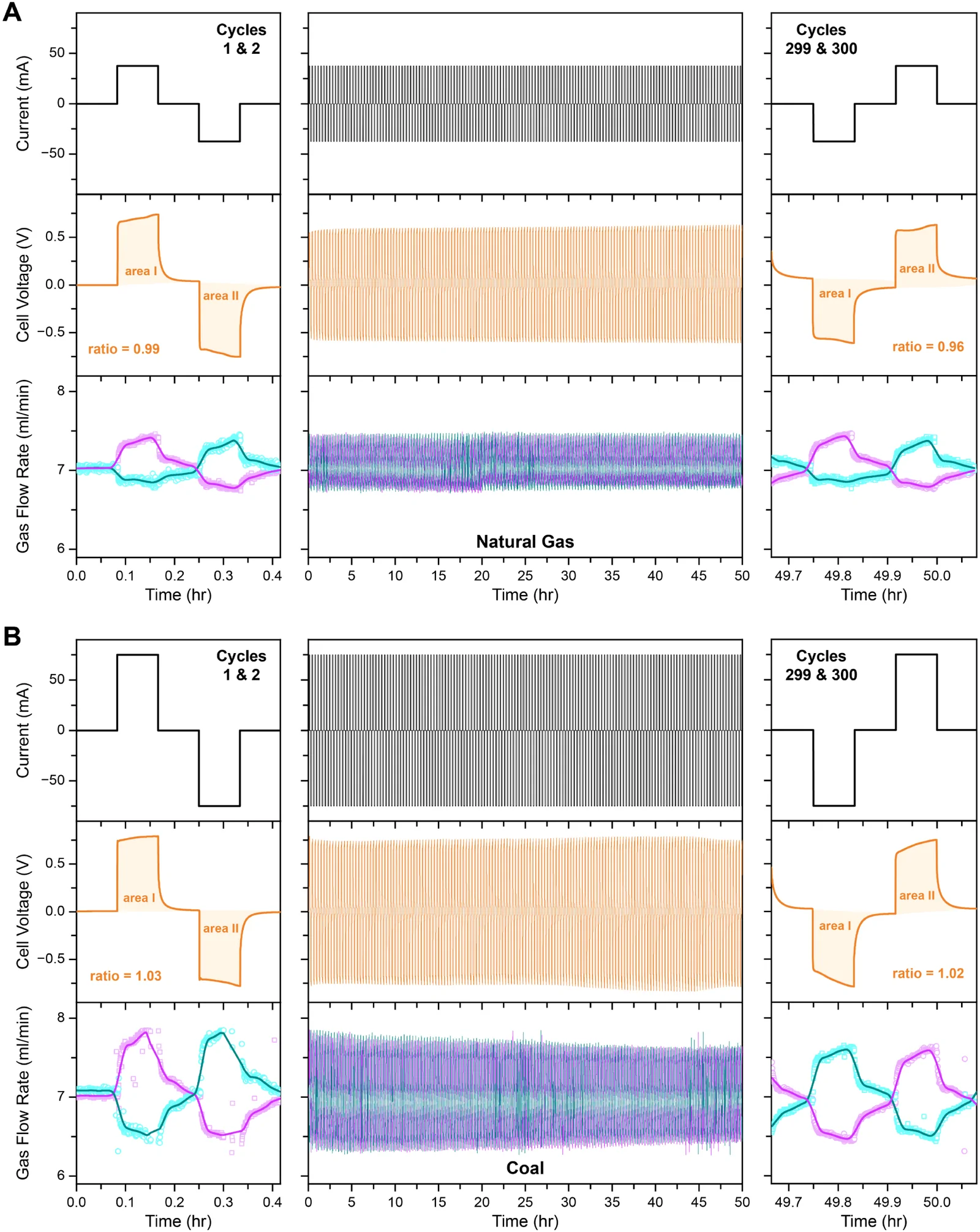
Long-term continuous operation under accelerated polarity-switching conditions. **(A, B)** Time-resolved response during a 300-cycle accelerated stability test conducted under optimized operating currents for (A) natural gas (37.5 mA) and (B) coal flue gas (75 mA). For each case, the applied current profile (top), corresponding cell voltage response (middle), and measured gas-phase CO_2_ flow rates associated with absorption at the cathode GDE and desorption at the anode GDE (bottom) are shown. The left panels highlight the first two cycles, the center panels display the full 300-cycle operation over 50 h, and the right panels show the final two cycles, enabling direct comparison of early- and late-stage behavior. Shaded regions in the voltage traces denote the integrated areas under positive and negative polarity segments, with the reported area ratios quantifying cycle-to-cycle reversibility.

To more quantitatively assess reversibility and long-term stability, the first two and last two cycles were examined in detail by comparing the ratio of the areas under the positive- and negative-polarity voltage segments. A ratio of 1 indicates ideal symmetry between consecutive cycles. For natural gas, the initial ratio was 0.99 and remained 0.96 after 300 cycles, demonstrating minimal voltage drift and strong operational stability. Coal exhibited similarly robust behavior, with ratios of 1.03 at the start and 1.02 after prolonged cycling. Taken together, these results confirm excellent cycle-to-cycle reversibility and high long-term stability even under accelerated testing conditions.

The long-term cycling results demonstrated that the two-stage EMAR system possesses the electrochemical robustness required for continuous deployment in fossil-fuel power plants supplying AI data centers. The near-unity voltage-area ratios observed across 300 polarity-switching cycles serve as a practical indicator of system durability: any appreciable electrode or electrolyte degradation would manifest as a measurable deviation from unity. Potential electrode degradation pathways include imbalanced rates of copper oxidation and reduction, irreversible surface passivation of the GDEs, loss of hydrophobicity, mechanical wear of the porous structure, or delamination of electrodeposited copper. Electrolyte degradation, primarily through amine breakdown, also represents a possible concern, though it is expected to be minimal under the ambient-temperature conditions of the two-stage EMAR process. A comprehensive suite of analytical and morphological measurements was conducted to assess these degradation pathways after 300 cycles. Scanning electron microscopy (SEM) was used to evaluate GDE morphology and identify any structural or surface changes. Titration and gas chromatography (GC) were employed to probe amine composition and potential formation of degradation products in both the liquid and gas phases. Ion chromatography (IC) and UV-vis spectroscopy analyses were used to detect any leached or newly formed species in the electrolyte, which could signal electrode deterioration or chemical degradation of the electrolyte. UV-vis analysis showed no major copper imbalance. GC did not detect any side product in the exit stream other than the gases at the inlet, i.e., N_2_, O_2_, and CO_2_; therefore, the process rules out any possibility of the hydrogen evolution reaction (HER) or CO_2_ reduction (CO2RR) that might lead to side reactions. These characterization results are detailed in Section S3 of the Supplementary Information. In summary, no measurable degradation was observed in either the GDEs or the electrolyte, consistent with the stable, near-unity voltage-area ratios throughout the 300-cycle test. Collectively, these findings validate the durability of the integrated GDE-based architecture and confirm its suitability for long-term, modular SMEC operation, providing a strong foundation for scaling to stacked cells, multi-module assemblies, and field-relevant deployment.

Energetics and desorption efficiency, which represents the Faradaic efficiency (FE) of the desorption, were quantified across all 300 cycles (Figure 5A). Under natural gas conditions, the desorption efficiency remained consistently high, fluctuating between ∼90% and ∼95%. Under coal conditions, the desorption efficiency gradually decreased from ∼92% to ∼82% before rising slightly to ∼84% by cycle 300. The origin of this gradual decrease is still under investigation; however, we hypothesize that transient local variations in CO_2_ availability at the anode GDE (arising from the higher CO_2_ concentration and faster reaction turnover under coal conditions) may introduce minor mass-transfer imbalances that accumulate over many cycles. The energetics remained stable for the natural gas case, ranging between 50 and 55 kJ_e_/molCO_2_. This stability is consistent with both the nearly constant cell voltage (Figure 4A) and the high, steady efficiency maintained across all cycles. For coal, the energetics increased modestly from ∼75 to ∼85 kJ_e_/molCO_2_, driven primarily by the gradual efficiency decline noted above. The higher energetics observed for coal relative to natural gas reflect the higher operational current (75 mA versus 37.5 mA), which increases the voltage load and thus the specific energy consumption. Nevertheless, the higher current also translated to a higher CO_2_ capture rate, and thus the increased energetics must be interpreted in the context of the greater throughput achieved under coal flue gas conditions.

**Figure 5:**
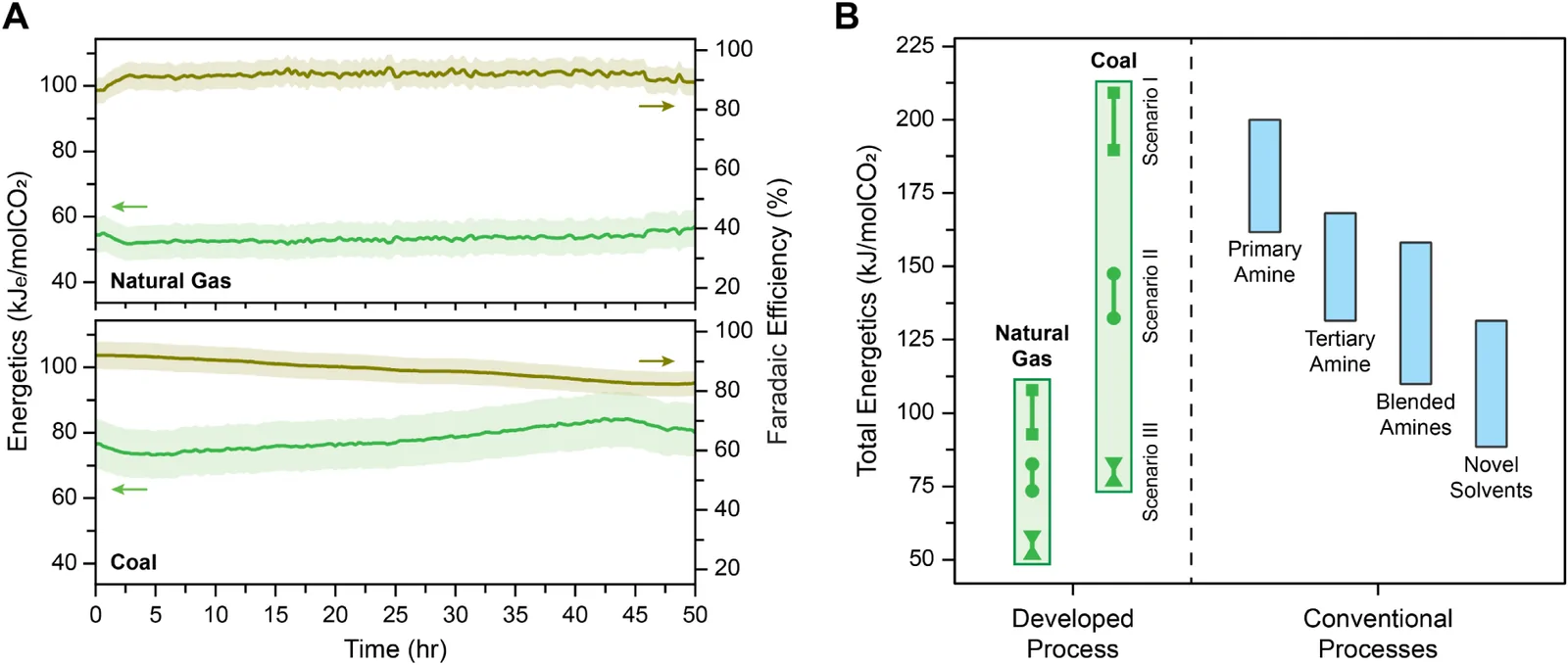
Long-term energetics and Faradaic efficiency and total energy comparison with thermal amine scrubbing. (A) Time-resolved evolution of electrical energetics (left axis, kJ_e_/molCO_2_) and Faradaic efficiency (right axis, %), calculated from the measured CO_2_ released at the anode relative to the theoretical charge-based CO_2_amount, measured over 300 continuous polarity-switching cycles for natural-gas (top) and coal-derived (bottom) flue gas compositions. (B) Comparison of the total primary-energy requirement (kJ/molCO_2_) for the developed two-stage EMAR process with conventional thermal amine scrubbing technologies. For the developed process, three electricity-sourcing scenarios are shown: *Scenario I*, electricity supplied entirely by the fossil-fuel power plant (accounting for generation efficiency penalties); *Scenario II*, a 50/50 mix of fossil-derived and renewable electricity; and*Scenario III*, electricity supplied exclusively by renewable sources. For each scenario, the vertical bars represent the full range of total energetics calculated from the minimum and maximum electrical energetics observed during long-term operation in panel A. Conventional benchmarks for primary amines (e.g., MEA), tertiary amines, blended amines, and emerging solvent systems are included for comparison.

#### 2.1.3 Comparison of Total Desorption Energetics with Conventional Thermal Amine Scrubbing

To contextualize the performance of the two-stage EMAR process, the total energetics required for CO_2_ capture were compared against those of conventional amine scrubbing with thermal regeneration (Figure 5B). The energy comparison in Figure 5B represents the cell-level energy requirement of the developed electrochemical process, converted to total primary energy based on the electricity source. Here, “total” refers to the energy supplied by the source and converted to the electrical energy required for CO_2_ desorption in the electrochemical cell. It does not include downstream CO_2_ concentration or compression, flue-gas delivery, and pressure-drop losses. Conventional systems, including primary amines, tertiary amines, blended amines, and emerging solvent formulations, typically report regeneration energies in the range of 80–200 kJ/molCO_2_,^46–49^ driven by the heat duty required for solvent stripping and reboiler operation. In contrast, the developed electrochemical process requires only electrical energy input. Because the nature of the energy input differs fundamentally between electrochemical and thermal systems, a direct comparison requires converting the electrical energy demand of the electrochemical system (kJ_e_/molCO_2_) into the total primary-energy requirement (kJ/molCO_2_) associated with generating that electricity. The total energetics of the electrochemical system depend on the source of electricity used to power the SMEC units, specifically, whether electricity is supplied by the fossil-fuel power plant itself or by co-located renewable sources (e.g., wind or solar). To bracket the plausible operating envelope for data-center energy infrastructures, three hypothetical sourcing scenarios were evaluated: *Scenario I:* Electricity sourced exclusively from the fossil-fuel power plant. In this scenario, the total energetics include the energy required at the plant to generate the electricity consumed by the SMEC units. The conversion efficiency of the generator, therefore, becomes a critical parameter. Representative generation efficiencies of 60% for natural gas combined-cycle plants and 35% for coal-fired plants were applied.^50,51^ Accordingly, the electrical energetics were divided by the appropriate efficiency to yield the total primary-energy requirement. *Scenario III:* Electricity sourced exclusively from renewable generation. When SMEC units are powered entirely by renewable electricity, the total energetics reduce to the measured electrical energetics, as no additional thermal input or conversion penalty applies. While idealized, this scenario provides a lower boundary for the achievable total energetics of the system. *Scenario II:* Electricity sourced from a 50/50 mix of power plant and renewable electricity. Because neither extreme case (Scenarios I and III) fully reflects real-world grid operation, an intermediate mixed-supply case (50% fossil, 50% renewable) was considered. This scenario provides a more representative estimate of practical operation at modern data-center energy campuses, where renewables are increasingly deployed but cannot fully satisfy the instantaneous load. In practice, determining the optimal electricity-sourcing strategy would require system-level grid optimization that accounts for plant dispatch, renewable intermittency, time-of-day pricing, curtailment events, and surplus generation. Such optimization is beyond the scope of the present study; therefore, Scenario II is adopted as a reasonable operational baseline against which to benchmark the total energetics of the electrochemical capture process.

For natural gas flue gas, all three sourcing scenarios (I–III) yielded total energetics in the range of ∼50–110 kJ/molCO_2_, which are substantially lower than those of conventional primary-amine systems (e.g., MEA), the established benchmark for point-source carbon capture (Figure 5B). The two-stage EMAR energetics were also consistently below those reported for tertiary amines and were comparable to or lower than values typical of blended-amine and next-generation solvent systems. For coal-derived flue gas, the total energetics for Scenarios II and III fell below the MEA benchmark and remained broadly comparable to other conventional solvent classes. Scenario I, which reflects the upper bound of total energetics due to the low conversion efficiency of coal-fired power generation, exceeded the MEA benchmark by a modest margin. These comparisons highlight that the developed electrochemical process delivers competitive, and in many cases substantially lower, total energetics relative to established thermal-swing technologies. Importantly, reduced energetics represent only one dimension of the advantage of the two-stage EMAR architecture. The combination of modularity and containerized deployment (in the form of SMEC), low-temperature operation that eliminates solvent degradation pathways, straightforward scalability through stack/module replication, and ease of retrofitting without large absorber columns collectively provide a systems-level benefit that conventional thermal amine scrubbing cannot offer. These attributes position the SMEC, with the two-stage EMAR as its core technology, as a compelling alternative for decarbonizing power plants serving AI data-center infrastructures. Having established the optimal operating conditions, long-term stability, and favorable energetics under idealized flue-gas compositions, the next critical question concerns its robustness under realistic operating environments where trace impurities are inevitably present.

To place these results in a broader context, the energetics of the developed process were also compared with those reported for recent ECC systems,^52–57^ as summarized in Section S4 of the Supplementary Information. Although process boundaries, feed compositions, and reporting conventions vary across the ECC literature, the developed process remains competitive with the most relevant recent reports and lies toward the lower end of the reported electrical-energy range for gas-fed, post-combustion-relevant ECC systems. This comparison further supports that the favorable energetics observed here are not only competitive with conventional thermal scrubbing but also with emerging ECC platforms.

### 2.2 Impurity Tolerance Under Realistic Flue Gas Conditions

While the major components of flue gas from fossil fuel-fired power plants, such as CO_2_, N_2_, and O_2_, are well characterized, trace impurities can potentially impact carbon capture performance. Among these, sulfur oxides (SOx) and nitrogen oxides (NOx) warrant careful consideration, particularly in electrochemical systems, where they may participate in parasitic chemical and/or electrochemical reactions that degrade long-term performance. Therefore, a thorough evaluation of these effects is critical for assessing the viability of electrochemical carbon capture technologies in real-world deployment. To date, the influence of such impurities has not been systematically investigated in the context of any electrochemical carbon capture process.

Here, we specifically investigate the impact of sulfur dioxide (SO_2_), the predominant SOx species, and nitric oxide (NO), the most common NOx species.^58^ Since these impurities are commonly present in flue gas from fossil fuel-fired power plants, the primary focus was on gas compositions representative of natural gas- and coal-fired power plants supplying electricity to data centers. Due to the safety and regulatory challenges associated with handling these gases experimentally, a detailed modeling approach was adopted. The model incorporates all relevant chemical equilibria and electrochemical pathways to fully capture the potential effects of these impurities on process chemistry and electrochemical performance. Full model formulation and assumptions are provided in Section S4 of the Supplementary Information. Accordingly, this section should be interpreted as a modeling-based impurity assessment, not as an experimental demonstration of SOx/NOx tolerance. Experimental validation under continuous flue-gas operation remains necessary to determine the effects of impurity exposure on SMEC performance.

#### 2.2.1 Sulfur Dioxide (SO_2_)

Similar to CO_2_, SO_2_ is an acid gas and can be absorbed by the high-pH electrolyte, potentially competing with CO_2_ and thereby reducing capture efficiency. To evaluate this effect, a comprehensive thermodynamic model was developed incorporating gas-liquid equilibria, acid–base reactions, and metal-ligand complexation (refer to Section S4.1 in the Supplementary Information). The analysis focused on SO_2_ concentrations relevant to both coal- and natural gas-fired power plants. For coal-derived flue gas, which typically contains higher levels of SO_2_, concentrations ranging from 2000 ppm down to 1 ppb were considered^59,60^ to assess both the direct impact of SO_2_ and the level of flue gas pre-treatment required to mitigate it. For natural gas-fired power plants, a range from 10 to 0.01 ppb was selected,^59,60^ reflecting the significantly lower SO_2_ content in these systems. Key outputs of the model as a function of SO_2_ concentration included electrolyte pH, concentration of redox-active species (i.e., Cu­(EDA)_2_^2+^), total absorbed CO_2_, both as carbamate (i.e., EDA-CO_2_) and dissolved inorganic carbon (DIC; i.e., [H_2_CO_3_] + [HCO_3_^–^] + [CO_3_^2–^]), and EDA speciation (Figure 6). These results collectively provide a comprehensive evaluation of how SO_2_ impurities at various concentrations affect the chemical and electrochemical environment of the capture system.

**Figure 6:**
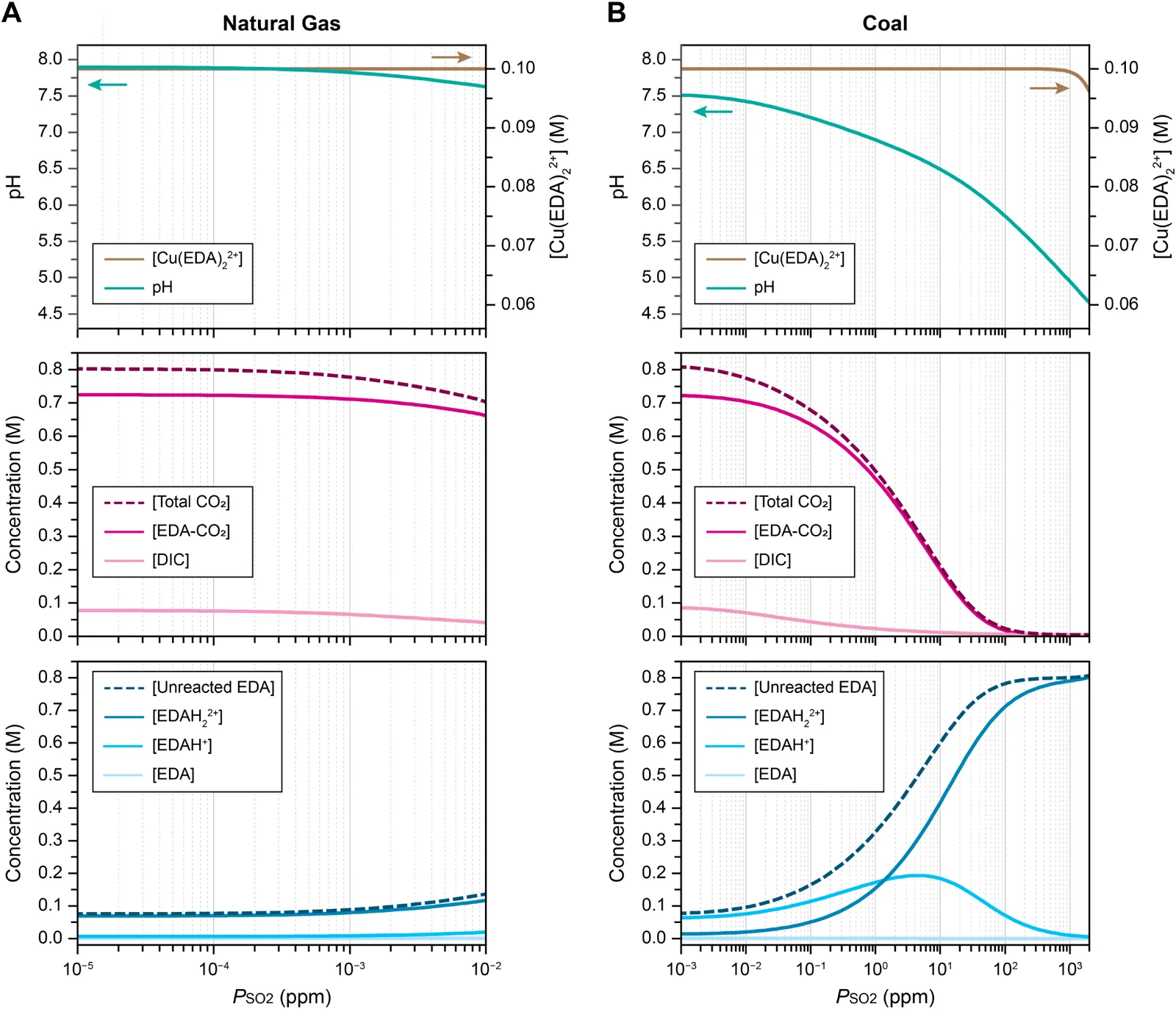
Impact of SO_2_ impurities on electrolyte speciation and CO_2_ capture performance. **(A, B)** Results of thermodynamic speciation modeling showing the influence of SO_2_ partial pressure (*P*_SO2_ in ppm) on key chemical variables for flue gas compositions representative of (A) natural gas-fired and (B) coal-fired power plants. **Top panels:** Predicted electrolyte pH (left axis) and concentration of the redox-active copper complex, Cu­(EDA)_2_^2+^(right axis), as a function of SO_2_ concentration. **Middle panels:** Total absorbed CO_2_ (dashed line), resolved into carbamate-bound CO_2_ (EDA-CO_2_) and dissolved inorganic carbon (DIC), where DIC = [H_2_CO_3_] + [HCO_3_^–^] + [CO_3_^2–^]. **Bottom panels:** EDA speciation, including unreacted EDA, which was defined as the sum of free and protonated amine species, i.e., [Unreacted EDA] = [EDA] + [EDAH^+^] + [EDAH_2_^2+^].

For coal-fired power plants, untreated flue gas, often containing more than 500 ppm of SO_2_, poses a significant challenge to the viability of the developed electrochemical carbon capture process (Figure 6B). At these concentrations, the electrolyte becomes highly acidified, with the pH dropping below 6. Despite this acidity, the concentration of the redox-active Cu­(EDA)_2_^2+^ complex remains largely unaffected, even at elevated SO_2_ levels. This resilience is attributed to the strong binding affinity between Cu^2+^ and EDA; at 2000 ppm of SO_2_, the Cu­(EDA)_2_^2+^ concentration is reduced by less than 4%. As a result, the electrochemical characteristics of the system, such as the voltage–current behavior, are not expected to be significantly impacted by SO_2_ under these conditions. In contrast, total CO_2_ absorption by the electrolyte is highly sensitive to SO_2_ content. Beyond 500 ppm of SO_2_, the system exhibits negligible CO_2_ uptake. This decline is primarily due to the suppression of carbamate formation, with a comparatively minor contribution from changes in DIC levels. Speciation analysis of the EDA absorbent reveals that the loss of CO_2_ capture capacity is primarily due to protonation reactions. At 500 ppm of SO_2_, 2% of total EDA exists as EDAH^+^ and 78% as EDAH_2_^2+^. Thus, approximately 80% of EDA that would otherwise participate in CO_2_ capture via the carbamate pathway is rendered unavailable (noted as “unreacted” in the figure) due to competitive acid–base equilibria. In all SO_2_ concentrations studied, 20% of EDA remains complexed with Cu^2+^. These findings highlight the necessity of flue gas pre-treatment. Conventional wet flue gas desulfurization (FGD) can reduce SO_2_ concentrations to approximately 10 ppm;^61,62^ however, even at this level, the carbon capture capacity of the process is reduced by ∼75%. Incorporating a secondary polishing scrubber to lower SO_2_ below 1 ppm can significantly improve performance,^63^ reducing capacity loss to approximately 30% (Figure 6B). The impact of SO_2_ in natural gas flue gas is notably different from that observed in coal-derived flue gas, primarily due to the significantly lower SO_2_ concentrations typically present (Figure 6A). Even at the upper bound of the investigated range, the effect on electrolyte pH is minimal, with values decreasing only slightly from 7.89 to 7.62. This modest acidification results in a negligible reduction in total CO_2_ absorption (∼10%). The carbamate pathway, which constitutes the dominant CO_2_ capture mechanism in this system, remains largely unaffected, exhibiting less than a 3% decrease. At the highest SO_2_ concentration considered, EDA speciation analysis shows that 11% exists as EDAH^+^ and approximately 2% as EDAH_2_^2+^, leaving only ∼13% of the total EDA pool unavailable (unreacted) for CO_2_ capture (Figure 6A). This result reflects a worst-case scenario at the upper limit of expected SO_2_ concentrations. Still, this loss is substantially lower than the ∼80% unavailability observed under coal flue gas conditions.

These results indicate that the successful implementation of the developed SMEC, with the two-stage EMAR as its core technology, for data center decarbonization at coal-fired power plants would require advanced desulfurization technologies to avoid severe performance degradation. Without such treatment, the presence of SO_2_ in untreated coal flue gas would substantially reduce CO_2_ capture capacity due to extensive protonation of EDA. Importantly, these desulfurization steps are not unique to the process; they are already standard practice in conventional amine-based carbon capture systems,^62,64^ and their adoption here would primarily involve system-specific integration and process customization rather than the development of entirely new pretreatment technologies. For natural gas power plants, by contrast, SO_2_ is expected to have a negligible impact on the electrochemical performance of the developed system under typical operating conditions, even in the absence of a dedicated SO_2_ removal unit.

It should also be noted that the present analysis is based on thermodynamic modeling and therefore reflects a conservative, worst-case assessment of SO_2_ impacts. In practical operation, kinetic limitations may mitigate the extent to which SO_2_ perturbs EDA speciation or suppresses carbamate formation. To fully quantify the true impact of SO_2_ on the performance, systematic controlled experiments are required to capture the interplay between thermodynamics and kinetics under realistic flow, mixing, and electrochemical conditions. Collectively, these considerations suggest that while SO_2_ management is essential for coal-based deployment, natural gas-based applications offer a more straightforward and cost-effective pathway for near-term decarbonization of AI-driven data centers, a point further examined in the following section.

#### 2.2.2 Nitric Oxide (NO)

Unlike SO_2_, NO is not an acid gas and is therefore chemically inert with respect to electrolyte speciation, behaving similarly to N_2_. The primary concern with NO lies in its possible electrochemical reduction at the cathode, where the flue gas is introduced. If this occurs, it could reduce the Faradaic efficiency (FE) of the copper deposition reaction from the Cu­(EDA)_2_^2+^ complex; a process that regenerates EDA and enables subsequent CO_2_ capture via carbamate formation. To evaluate the potential impact of trace NO on cathodic FE, a coupled Butler–Volmer and mass-transport model was developed, incorporating five competing half-reactions, including the desired copper deposition reaction and undesired hydrogen evolution reaction or NO reduction via various pathways. The model was used to calculate FEs across a range of NO concentrations (200–1000 ppm), representative of flue gas compositions from both natural gas and coal-fired power plants.^59,60^ FE calculations followed the methodology detailed in Section S4.2 of the Supplementary Information.

Across the full range of NO concentrations studied, copper deposition consistently dominated the cathodic process, with FEs exceeding 99.5%. Hydrogen evolution accounted for the remaining ∼0.5%, while NO reduction currents were negligible and insensitive to increasing NO concentration in both flue gas scenarios. Given that NO reduction is thermodynamically favored under acidic conditions, further simulations were performed under electrolyte conditions corresponding to extreme SO_2_ contamination (which results in pH < 6). Even under these conditions, copper deposition remained the dominant pathway, with FEs above 98%. These modeling results are fully consistent with experimental observations reported in prior studies of EMAR,^37–40^ which have repeatedly demonstrated that copper deposition is the dominant cathodic redox reaction under practical operating conditions, with FEs measured to be near unity. These findings confirm that, under realistic kinetic and mass-transport constraints, NO does not appreciably compete with copper deposition at the cathode, even at concentrations as high as several hundred ppm. Therefore, NO can be considered not only chemically but also electrochemically inert within the context of the developed system. As a result, mitigation of NO in the flue gas may be deprioritized relative to more disruptive impurities such as SO_2_. In continuous operation, SO_2_ cannot be treated only as a one-time impurity because absorbed sulfite/sulfate species could accumulate in the electrolyte if no purge or pretreatment is used. A practical SMEC system would therefore require upstream desulfurization for coal-derived flue gas, together with electrolyte monitoring of pH, conductivity, sulfate/sulfite concentration, and alkalinity measurement for cyclic CO_2_ capacity. If accumulation occurs, a make-up stream strategy that normally is utilized in gas sweetening must be followed to maintain solvent performance. However, NO is less likely to accumulate in a way that affects the cycle because it is not an acid gas and is predicted to have negligible electrochemical activity in the given modeled conditions.

### 2.3 Case Study: Deployment of SMEC for AI Data Center Decarbonization

To evaluate the practical feasibility of the developed SMEC system for data center decarbonization, a hypothetical implementation scenario was constructed for OpenAI’s largest planned data center, Stargate, currently under development in Abilene, Texas. This facility, among the largest AI data centers globally, is designed to accommodate up to 400,000 NVIDIA GB200 (Blackwell) GPUs. Once fully operational, the data center is projected to require approximately 1.2 GW of electrical capacity. Power is expected to be supplied through a combination of renewable sources, including West Texas wind farms supported by large-scale battery storage and an on-campus solar installation for daytime loads, and a 360 MW on-site natural gas power plant. While wind and solar are considered low-carbon sources, the natural gas component introduces a substantial carbon footprint and necessitates carbon capture to achieve full decarbonization of the facility. Based on a standard emissions factor of 0.4 tonnes CO_2_ per MWh of electricity generated^65,66^ and a typical 60% capacity factor, the on-site natural gas plant is estimated to emit approximately 760,000 tonnes of CO_2_ annually.

To assess the deployment logistics and economic implications of SMEC integration, the required number of SMEC units was estimated for three representative decarbonization scenarios: 98% capture (denoted as the “*ideal*” case, reflecting the upper limit of carbon capture performance), 90% capture (the “*typical*” case for commercial carbon capture systems), and 50% capture (the “*partial*” decarbonization case). For each scenario, the corresponding impact on the levelized electricity cost from the natural gas power plant was also calculated. Full technical and economic details, along with all underlying assumptions, are provided in Sections S5 and S6 of the Supplementary Information. This analysis is a deployment framework based on single-cell performance and scale-up assumptions. It does not represent an experimentally demonstrated container-scale SMEC unit.

In each case, two key design parameters associated with the electrochemical cell were systematically evaluated to capture the potential effects of materials development and system optimization: the electrochemical active surface area to geometric area ratio (ECAS-to-GA), and the applied current density. The ECAS-to-GA parameter quantifies the enhancement of the available surface area for gas-liquid contact and electrochemical reactions due to the use of porous, high-surface-area GDEs. The current density governs the rate of CO_2_ capture and directly impacts the throughput of each electrochemical module. Two representative design configurations were investigated: (i) ECAS-to-GA of 8 and a current density of 200 A/m^2^, representing the “*current*” capabilities of SMEC systems employing commercially available carbon-based GDEs; and (ii) ECAS-to-GA of 12 and a current density of 500 A/m^2^, reflecting a “*future*” advancement in GDE microstructure and mass transport optimization, and consistent with current densities typical of stationary polymer electrolyte membrane (PEM) fuel cells and electrolyzers.^67,68^ The future projection is informed by historical development trajectories of PEM fuel cells and water electrolyzers, which share inherent structural and operational similarities with SMEC systems.

#### 2.3.1 Required Number of SMEC Units

The number of SMEC units required to achieve targeted levels of decarbonization for the Stargate data center was estimated under the aforementioned three electrochemical performance configurations (*current*-state, *modest* advancement, and *future* development). Detailed information on SMEC packaging design and system configuration, including module count, stack architecture, spatial arrangement, and associated engineering specifications, is provided in Section S5 of the Supplementary Information. Under the *ideal* decarbonization scenario (98% CO_2_ removal), the *current*-state configuration requires approximately 19.9 SMEC units. With *future* improvements in ECAS-to-GA and current density, the number of units decreases to 5.3. For the *typical* decarbonization scenario (90% CO_2_ removal), the required number of SMEC units is reduced to 18.3, and 4.9 for the *current* and *future* configurations, respectively. In the case of *partial* decarbonization (50% CO_2_ removal), the system requirements are further reduced to 10.2 and 2.7 units, respectively, across the same performance tiers (Figure 7A). These results highlight that even under current technological constraints, the deployment requirements remain practical and highly promising. With fewer than 20 40-foot SMEC containers required to achieve nearly complete decarbonization of a 360 MW natural gas power plant, the spatial footprint of the system is remarkably compact. Assuming a 1:1 ratio of SMEC units to ancillary equipment (e.g., compressors, balance-of-plant), the total estimated footprint would occupy less than 0.01% of the Stargate campus area of ∼875 acres. Therefore, spatial constraints are unlikely to pose a meaningful limitation to the integration of this technology at scale, and future technological improvements will primarily reduce capital costs by lowering the number of SMEC units required.

**Figure 7:**
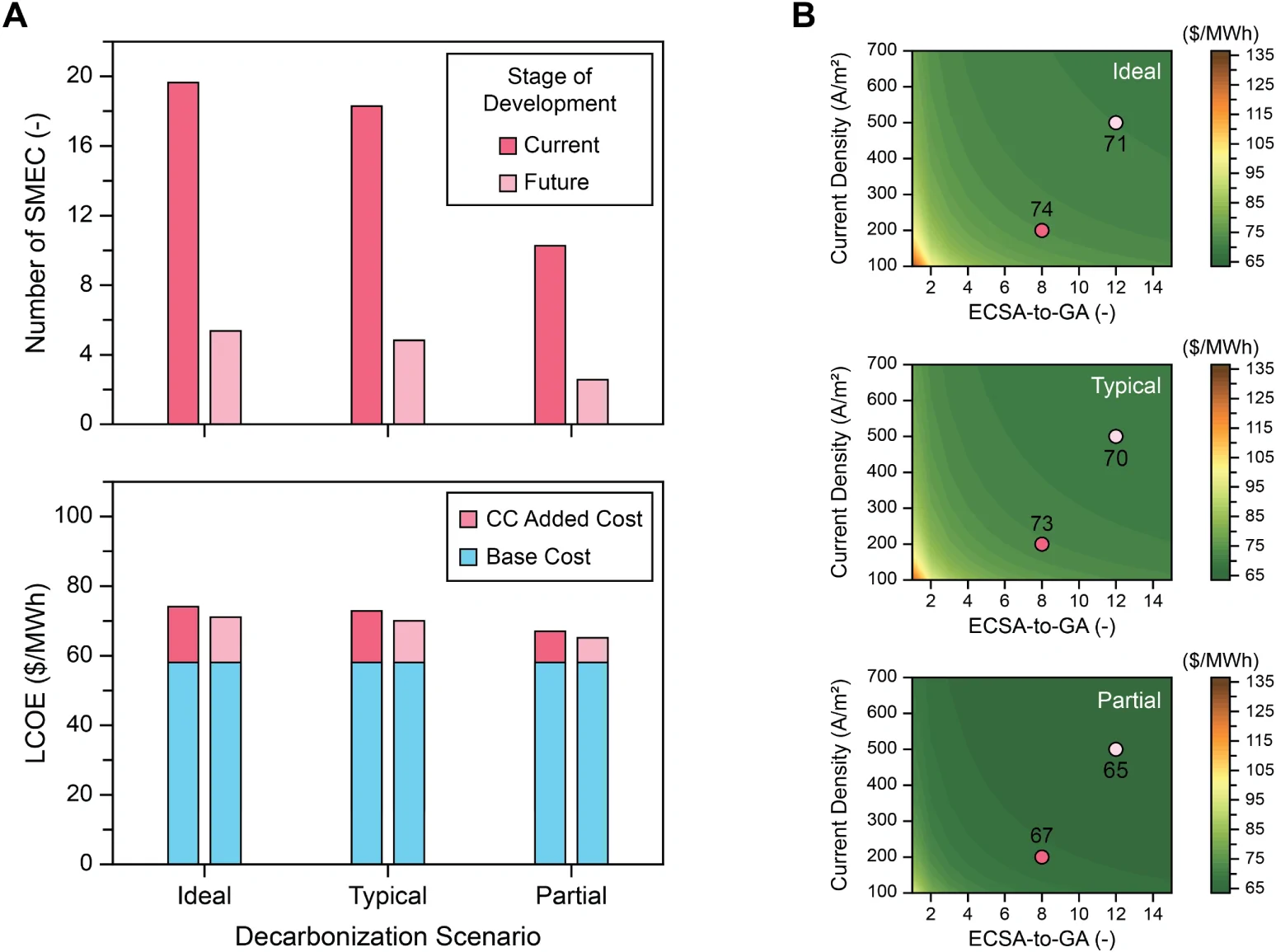
Economics of SMEC Deployment. (A) Number of required SMEC units (top panel) and levelized cost of electricity (LCOE) (bottom panel) for the*ideal* (98%), *typical*(90%), and*partial* (50%) decarbonization scenarios, calculated for*current* and *future* stages of development. LCOE was calculated by incorporating the additional costs related to the carbon capture (CC) unit into the baseline electricity cost of $58/MWh. (B) LCOE estimation across broader ranges of ECAS-to-GA ratio and current density to further assess the impact of technology development, calculated for the*ideal* (top panel), *typical* (middle panel), and*partial* (bottom panel) decarbonization scenarios. The two circle symbols represent the LCOE associated with the*current* (dark pink) and*future* (light pink) stages of development.

#### 2.3.2 Techno-Economic Evaluation of SMEC Integration

Integrating a carbon capture system into a natural gas power plant inherently increases the levelized cost of electricity (LCOE) due to added capital and operating expenditures. To quantify this impact, a comprehensive TEA was performed to assess the cost implications of deploying the SMEC system under various decarbonization targets and technology development scenarios. The TEA framework builds upon our previously established methodologies for electrochemical systems, incorporating detailed cost modeling for capital investment, operational expenditures, system lifetime, and module-level balance of plant,^69^ with SMEC-specific parameters adapted accordingly. Additional considerations included capacity factor assumptions, labor, maintenance, and compression costs for CO_2_ conditioning.

In West Texas, where the Stargate data center is being constructed, the average LCOE for a natural gas combined-cycle power plant is approximately $58/MWh. If *ideal* decarbonization is pursued using SMECs at the *current* stage of development, the added cost from the carbon capture system is estimated to be $16/MWh, resulting in a total electricity cost of $74/MWh delivered to the data center. For the *typical* capture scenario, the additional carbon capture-related cost ranges from $15/MWh (total LCOE of $73/MWh) under the *current* configuration to $12/MWh (total LCOE of $70/MWh) under the *future* development case. For *partial* decarbonization, LCOE increases to $67/MWh under the *current* configuration and to $65/MWh under the *future* development (Figure 7A). Notably, unlike the number of SMEC units, which decreases sharply with *future* improvements in electrochemical performance, the LCOE is comparatively insensitive to these advances. Even in the most conservative case of full decarbonization under *current*-state performance, the total LCOE increases by ∼22%, indicating that the dominant cost driver remains the baseline electricity generation rather than the incremental cost of carbon capture. Beyond the *current* and *future* design configurations, a broader parametric range of ECAS-to-GA and current density was evaluated across the three decarbonization scenarios to assess the sensitivity of LCOE to electrochemical performance (Figure 7B). This analysis provides a broader perspective on how the LCOE evolves if higher electrochemical performance metrics are achieved through future development or if the system does not fully maintain its current performance levels. Full technical assumptions and cost calculation methodologies are detailed in Section S6 of the Supplementary Information.

While the integration of carbon capture technology imposes an economic premium, this cost must be weighed against the broader imperative for sustainable AI infrastructure. Large-scale AI models require immense energy input, and their carbon footprints are under increasing scrutiny.^7^ Incorporating carbon capture solutions like SMEC provides a direct pathway for reducing emissions associated with AI computing, helping align data center operations with corporate sustainability targets, investor expectations, and forthcoming regulatory pressures on digital infrastructure emissions. This analysis is a deployment framework based on single-cell performance and scale-up assumptions. Stack, module, and container-scale performance has not yet been experimentally validated, and the reported SMEC deployment scenarios should therefore be interpreted as scale-up projections.

## 3 OUTLOOK

This study addressed the growing challenge of decarbonizing fossil-fuel power plants that support energy-intensive AI data center infrastructure. We introduced SMECs as a new, deployment-oriented class of electrochemical carbon capture technology specifically designed to meet the spatial, economic, and operational constraints of AI power systems. By coupling containerized modularity with an electrochemical process that operates at ambient conditions, this study demonstrated a pathway for delivering low-carbon electricity without compromising the reliability required by large-scale AI computing.

At the core of the SMEC architecture, a two-stage membraneless EMAR process was evaluated at the single cell level. By integrating CO_2_ absorption and electrochemical desorption within a single compact electrochemical cell, this design eliminated absorber columns and ion-exchange membranes, enabling true plug-and-play modularity. Single cell operation was demonstrated under realistic flue gas compositions representative of natural gas- and coal-fired power plants. Polarization mapping identified optimal operating currents for both natural gas and coal scenarios, followed by a 300-cycle, 50-h accelerated polarity-switching test. Stable cycling, near-unity Faradaic efficiency, and competitive electrical energetics relative to conventional thermal amine systems were maintained throughout, supporting the electrochemical robustness of the integrated GDE-based architecture. No abnormal dendritic growth or electrode bridging was observed after 300 cycles by SEM, and the voltage-area ratio remained close to unity. Longer operation could still introduce dendrite formation or localized copper growth that may bridge the electrode gap if the electrodes are placed closer in an advanced design. Inconsistent copper growth could result from current collector failure, leading to uneven copper deposition. Therefore, voltage-profile monitoring during operation would help identify such inconsistencies and would require immediate corrective steps to restore consistent copper deposition.

The effects of flue-gas impurities were evaluated through a modeling-based assessment of SO_2_ and NO, which are critical for real-world deployment. Thermodynamic and electrochemical modeling showed that SO_2_ can significantly suppress CO_2_ capture in coal-derived flue gas through electrolyte acidification, establishing the need for advanced desulfurization in coal applications. In contrast, NO was shown to be both chemically and electrochemically benign, with copper deposition remaining the dominant cathodic reaction even under acidic conditions. These findings provided clear, process-specific guidance for pretreatment requirements and reduced uncertainty around impurity impacts in electrochemical capture systems.

To assess deployment feasibility at scale, a case study was conducted for OpenAI’s Stargate data center in Abilene, Texas. Fewer than 20 SMEC units were required to achieve near-complete decarbonization of the on-site 360 MW natural gas power plant, which was estimated to emit ∼760,000 tonnes CO_2_/yr. Despite significant reductions in the number of SMEC units achievable through future improvements in electrochemically active surface area and current density, the levelized cost of electricity remained comparatively insensitive to these advances. Even in the most conservative scenario of full decarbonization under current performance assumptions, the LCOE increased by only ∼22%, indicating that the dominant cost driver remains the baseline electricity generation rather than the incremental cost of carbon capture.

Several future opportunities for advancing the SMEC platform were identified. The impurity tolerance analysis relied on conservative thermodynamic and electrochemical modeling and should be complemented by controlled experimental validation under realistic flow, mixing, and electrochemical operating conditions. While accelerated long-term cycling demonstrated strong electrochemical stability, extended field operation under continuously varying flue-gas compositions remains necessary to fully assess durability at deployment time scales. In addition, although system-level performance was evaluated, further optimization at the stack and balance-of-plant levels, including compression, thermal management, and power electronics, will be required to minimize auxiliary energy penalties. Finally, grid-integrated operation and dispatch-aware control strategies were not addressed here and represent an important next step for deployment within dynamic data-center energy systems. Additional testing should include stack/module validation, longer operation, direct outlet-amine monitoring, and controlled impurity-spiked experiments. While the 300-cycle, 50-h accelerated polarity-switching test demonstrated stable electrochemical cycling, extended operation under real flue-gas conditions remains necessary to assess durability at deployment time scales.

Looking forward, continued advances in electrode materials via GDE microstructure engineering are expected to substantially reduce the number of SMEC units required. Operation at higher current densities, informed by progress in related electrochemical energy technologies, offers a clear pathway toward lower capital intensity and higher throughput per module. These improvements, coupled with stack-level scaling and containerized integration, define a realistic trajectory toward pilot-scale demonstrations and commercial field trials. Taken together, this work positioned SMECs as a scalable and flexible carbon capture architecture capable of supporting the sustainable growth of energy-intensive AI infrastructure while preserving the reliability advantages of fossil-fuel power generation.

## 4 MATERIALS and METHODS

The electrochemical tests were performed in a membraneless electrochemical cell employing gas flow channels on both the anode and cathode, supported by two GDEs.^45^ The GDEs were fabricated from carbon paper (Toray 060) and subsequently electrodeposited with copper using a method similar to that described in our earlier proof-of-concept study.^45^ Circular Toray 060 carbon papers with a radius of 2.3 cm were electrodeposited, with an active copper area of approximately 10.80 cm^2^ for current-density calculations. Electrodeposition was carried out in a standard copper bath solution at a fixed current of 200 mA for 45 min, resulting in a uniform copper loading of approximately 1.7 mg/cm^2^ on the GDE surface. This loading was found to be the optimum condition based on our previous study.^45^ The electrolyte solutions corresponding to natural gas and coal combustion scenarios were pre-purged with simulated flue gas mixtures (CO_2_, N_2_, and O_2_) representative of each case. The aqueous electrolyte consisted of 0.25 M copper­(II) sulfate pentahydrate (CuSO_4_·5H_2_O; 98% purity, Sigma-Aldrich) as the initial source of cupric ions (Cu^2+^), 1 M ethylenediamine (EDA; 98% purity, Sigma-Aldrich) as the amine absorbent, and 0.5 M sodium sulfate (Na_2_SO_4_; purity >99.0%, Sigma-Aldrich) as the background electrolyte to enhance solution conductivity. All solutions were prepared using deionized water with a resistivity greater than 18 MΩ·cm. A fixed volume of 50 mL of the electrolyte was added to a 100 mL three-neck round-bottom flask and stirred continuously at 300 rpm while purging with the respective flue gas for 2 h to achieve pre-saturation when needed.

The pre-saturated electrolyte was then transferred to the electrolyte chamber in the assembled cell and sealed with a stopper to ensure gas-tightness. The flue gas flow rate was maintained at 7 SCCM on both the anode and cathode sides. For the natural gas scenario, the flue gas composition consisted of 5% CO_2_, 13% O_2_, and 82% N_2_, while for the coal combustion scenario, the CO_2_ concentration was increased to 13%, with 4% O_2_ and 83% N_2_. These compositions were achieved by controlling the individual gas flow rates of the respective gases using mass flow controllers (DPCS, Aalborg) installed upstream, which were initially calibrated for each specific gas. Gas flow meters (XFMS, Aalborg) were installed downstream of both the anode and cathode to continuously monitor changes in gas flow rates during the absorption and desorption. For the coal scenario, the polarization range was extended from 25–125 mA (23–116 A/m^2^) with increments of 25 mA to account for the higher CO_2_ concentration, while for natural gas, this was 25 to 75 mA (23–69 A/m^2^) with increments of 12.5 mA. Current was applied using a potentiostat (VMP 300, Biologic) across the cell without a reference electrode, and the resulting cell voltages were recorded. Simultaneously, changes in gas flow rates were continuously logged on both sides of the cell using software (XFM communication utility, Aalborg).

The volumes of gas absorbed and desorbed during the negative and positive polarization stages of each cycle were calculated by integrating the deviation from the baseline flow rate of 7 SCCM. The amount of desorbed CO_2_ (in moles) was then used to calculate the energetics of desorption for each cycle according to (eq 1):

1 $$W=\frac{\int_{0}^{t}UIdt}{\Delta n_{desorbed}}$$

where *W* (kJ_e_/mol CO_2_) represents the electrical energetics, *U* (V) the derived voltage, and *I* (A) is the applied current. Δ*n_desorbed_* represents the moles of the CO_2_ desorbed.

Absorption efficiency, reported as SPE, was calculated by dividing the measured CO_2_ uptake at the cathode by the total inlet CO_2_ supplied to the cathode during the same absorption period. Desorption efficiency, reported as FE, was calculated by comparing the experimentally measured moles of CO_2_ released at the anode with the theoretical moles of CO_2_ expected from the charge passed.

The desorption efficiency (η, %) is calculated by comparing the moles of the CO_2_ desorbed experimentally to the theoretical moles of the CO_2_ (eq 2).

2 $$\eta_{desorption}=\frac{\Delta n_{desorbed}}{\Delta n_{theoretical}}\times 100$$

The theoretical moles of CO_2_ (Δ*n_theoretical_*) were calculated using (eq 3).

3 $$\Delta n_{theoretical}=\frac{Q}{F}=\frac{It}{F}$$

Here, *Q* (C) is the charge transferred during the experiment time *t* (s), and *F* (96,485 C/mol) is the Faraday constant. This calculation is based on the stoichiometric ratio of electrons to CO_2_ of 1:1, meaning that the transfer of one mole of electrons theoretically results in the desorption of one mole of CO_2_.

For long-term continuous operation, the electrolyte and flue gas composition, and data logging procedures were kept the same as described earlier for both the natural gas and coal combustion scenarios. The current, however, was fixed at the value determined by the polarization behavior, resulting in the optimal energetics and the efficiency for the respective scenario.

## Supplementary Material



## Notes

Membraneless EMAR Process and Background, Electrochemical Characterizations, Post-Cycling Electrode and Electrolyte Stability Analyses, GC/IC/UV-vis Characterization, Energetics Comparison, SO_2_ and NO Impurity Modeling, SMEC Packaging and Power-Plant Integration Framework, Techno-Economic Analysis (TEA) (PDF)
