# Supplementary material for: Small Modular Electrochemical Containers for Decarbonization of AI Data Centers
Source: Environ Sci Technol. 2026 Oct 29;60(44):31446–62. doi: 10.1021/acs.est.6c06512 (PMC13757946; doi:10.1021/acs.est.6c06512)
Supplement: Supplementary file 1 [file es6c06512_si_001.pdf]

SUPPORTING INFORMATION

for

Small Modular Electrochemical Containers for Decarbonization of AI Data Centers

Ahmad Hassan[1], Prince Aleta[1], Mohsen Afshari[1], Abdelrahman Refaie[1], Mim Rahimi[1,2,*]
[1] Department of Civil and Environmental Engineering, University of Houston, Houston, TX 77204, USA
[2] Materials Science and Engineering Program, University of Houston, Houston, TX 77204, USA
* Corresponding Author: mrahimi@uh.edu

Total pages: 39 pages, numbered S1-S39
Total figures: 8 figures, Figures S1-S8
Total tables: 9 tables, Tables S1-S9

Table of Contents

S1. EMAR DEVELOPMENT 2
S1.1. EMAR Process Scheme 2
S1.2. Recent Advancements of the Process 4
S2. FUNDAMENTAL ELECTROCHEMICAL CHARACTERIZATIONS 6
S2.1. Cyclic Voltammetry 6
S2.2. Electrochemical Impedance Spectroscopy 8
S3. ELECTRODE & ELECTROLYTE STABILITY AFTER LONG-TERM CYCLING 10
S3.1. Morphological Characterization of GDEs After 300 Cycles 10
S3.2. Electrolyte Composition and Amine Stability Analysis 12
S3.3. Trace Species Detection via IC and UV-Vis 14
S4. COMPARISON OF ENERGETICS WITH OTHER ECCs 17
S5. MODELING FRAMEWORK FOR SO_2 & NO IMPURITY EFFECTS 19
S5.1. Thermodynamic Modeling Framework for SO_2 19
S5.2. Kinetic model for NO reduction and Faradaic efficiency analysis 21
S6. SMEC PACKAGING DESIGN & POWER-PLANT INTEGRATION ANALYSIS 25
S6.1. SMEC Packaging and System Configuration 25
S6.2. Power-Plant Case Study and SMEC Fleet Sizing Framework 28
S7. TECHNO-ECONOMIC ANALYSIS 31
S8. SUPPLEMENTARY REFERENCES 36

S1. EMAR DEVELOPMENT

S1.1. EMAR Process Scheme

The original electrochemically mediated amine regeneration (EMAR) process is a typical four-stage process in which two of the stages involve chemical reactions, while the other two are electrochemical in nature (Figure S1A). The process begins with typical absorption in an absorber column, where the flue gas containing CO_2 and the amine solvent, ethylenediamine (EDA) interacts countercurrently to create carbamate ($EDA_{(aq)} + CO_{2\,(g)} \rightarrow EDA\text{–}CO_{2\,(aq)}$), while other gases (i.e., N_2 and O_2) leave the system as treated gas. The solution saturated with carbamates (i.e., $EDA\text{–}CO_{2\,(aq)}$) is pumped into the electrochemical cell, which consists of anodic and cathodic chambers separated by an anion exchange membrane (AEM). The anode and cathode are made of solid copper plates to serve as the active electrodes. The carbamates pumped to the anode chamber undergo a complexation reaction ($Cu^{2+}_{(aq)} + 2EDA\text{–}CO_{2\,(aq)} \rightarrow Cu(EDA)_2^{2+}{}_{(aq)} + 2CO_{2\,(g)}$) with Cu^{2+}, generated by oxidation of the copper anode ($Cu^0_{(s)} \rightarrow Cu^{2+}_{(aq)} + 2e^-$). The combination of the two reactions results in a two-phase mixture, with deactivated amine as the liquid and the CO_2 as the gas phase. This solvent deactivation by copper oxidation is called the second stage. Unlike the second stage, the third stage is chemical, where the flash tank achieves desorption of the CO_2 as pure gas, which is later concentrated and compressed either for further use or sequestration. However, the fourth stage corresponds to the reactivation of the amine. It's achieved in the cathodic chamber, where an electrochemical reaction ($Cu(EDA)_2^{2+}{}_{(aq)} + 2e^- \rightarrow Cu^0_{(s)} + 2EDA_{(aq)}$), deposits copper back at the cathode and results in the reactivation of the amine. The process continues until the anode is reasonably depleted. At that point, the polarity of the electrodes is switched (making the anode work as the cathode and vice versa) to reverse the changes that occurred at both the anode and cathode.

The membraneless EMAR process, on the other hand, not only removes the membrane from the process but also converts it into a two-stage process (Figure S1B). In this approach, the solid copper electrodes are replaced with gas diffusion electrodes (GDEs) for the exchange of gases; however, they still contain a thin layer of copper to carry out the electrochemical reactions. Stages 1 and 4, which in the original EMAR were specific to absorption of CO_2 and reactivation of the amine, respectively, are combined into a single stage. This stage is referred to as cathodic absorption, in which, as the amine is reactivated at the GDE interface, CO_2 from the flue gas passes gets selectively absorbed into the amine, thereby eliminating the need for an external absorber column. However, the second stage is the combination of traditional stages 2 and 3,

now called anodic desorption. Here, as soon as CO_2 is desorbed by the complexation reaction of amine with the Cu^{2+}, it is ready to leave the cell at the anode GDE, which previously required a flash tank for separation. Since the copper redox reaction is unchanged, a similar polarity-switching mechanism is required for continuous operation. However, a gas-switching four-way valve is required upstream to operate in sync with polarity switching by maintaining flue gas and sweep gas flows specific to the cathode and anode, respectively.

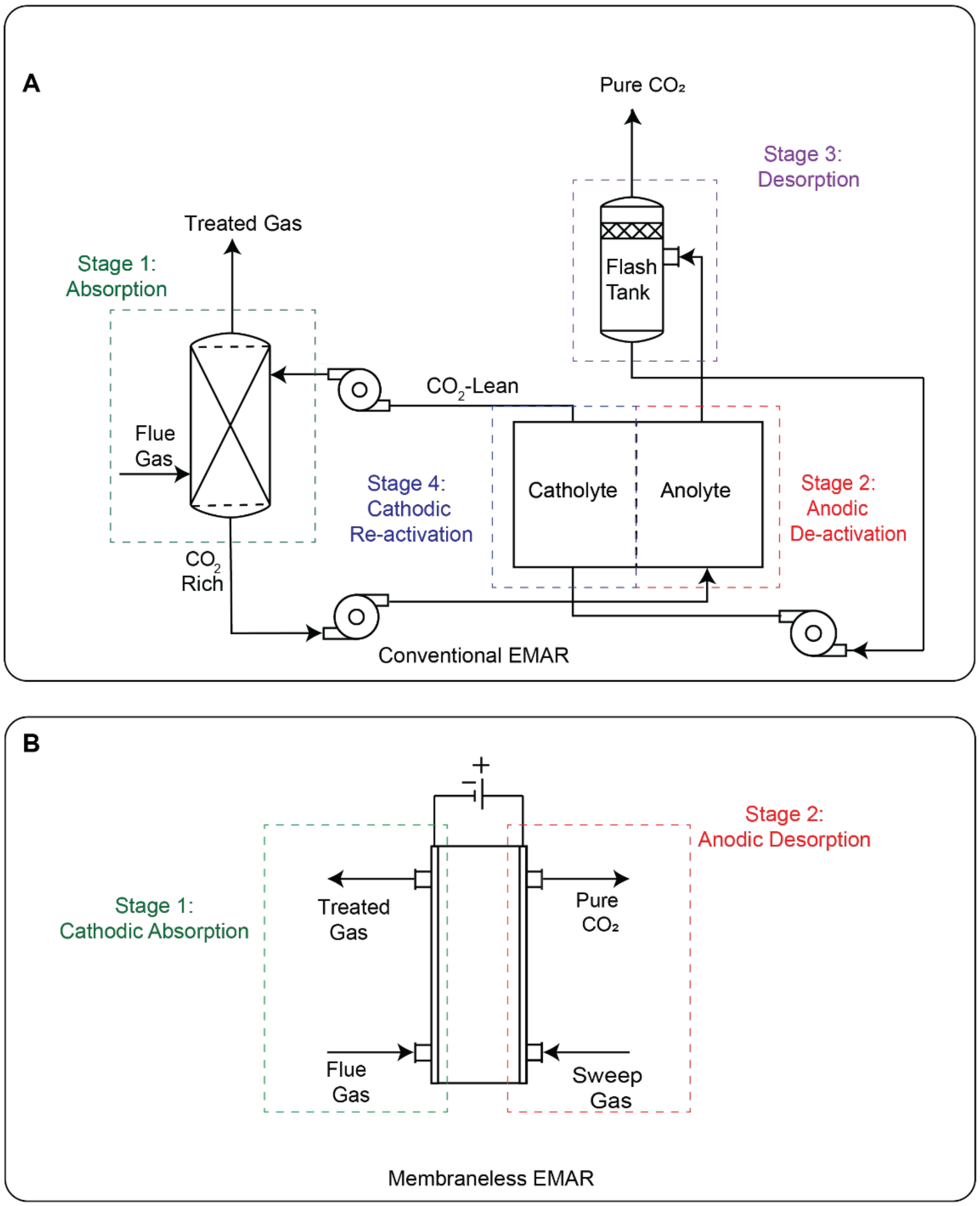

Figure S1. Process flow diagrams of the four-stage and two-stage EMAR. (A) conventional EMAR representing a four-stage process starting from the absorption of the CO_2 in the absorber to the removal of CO_2 as a pure stream. (B) Membraneless EMAR shows the two-stage process by combining the stages of the conventional EMAR. The electrode in membraneless EMAR is GDE rather than the solid electrode usually used in conventional EMAR.

S1.2. Recent Advancements of the Process

The transition from thermal to electrochemical carbon capture using amine began with the validation of the EMAR cycle, establishing that an electrical swing could effectively replace energy-intensive thermal regeneration while operating isothermally to mitigate amine volatility [1-3]. While several metal and amine combinations were initially screened to demonstrate feasibility, the copper and EDA couple was selected due to the high stability of the resulting complex and favorable redox thermodynamics. This chemistry has remained the reference for most subsequent studies [4]. Early bench-scale studies reported the energetics of 100 $kJ_e/molCO_2$ with Faradaic efficiencies of up to 80% at 50 A/m^2 [1, 2]. Continuous-flow operation was later demonstrated for more than 100 hours [5, 6], enabling detailed thermodynamic modeling at a broad range of copper loadings and CO_2 partial pressures across four stages [7]. Later, techno-economic analysis (TEA) based on this framework identified membrane cost as the dominant contributor to capital expenditure (CapEx), while indicating that operation at approximately 80% copper loading was near-optimal for efficient CO_2 removal [8].

Building on the copper-EDA framework established in early EMAR studies, subsequent studies pointed out physical and chemical limitations that directly affected the efficiency. At higher current densities, gas evolution at the anode became a dominant operational challenge, as accumulated CO_2 bubbles blocked the active copper surface, increased Ohmic resistance, and destabilized the cell voltage. This limitation was addressed by introducing ionic surfactants, sodium dodecyl sulfate (SDS), into the electrolyte to modify surface tension and promote the formation of smaller, rapidly detaching bubbles. This intervention stabilized electrode operation and reduced energy consumption by approximately 25% [9-11]. In parallel with interfacial bubble release optimization, the absorbent chemistry evolved to address solvent volatility and reduce regeneration energy. Early formulations focused on practical handling by blending EDA with aminoethylethanolamine (AEEA) to suppress vapor pressure, which reduced solvent loss but did not change the underlying capture mechanism [5]. A more practical advancement came from recognition that efficient EMAR operation does not require the entire amine to be electroactive. Blends of electroactive EDA with non-electroactive amines such as methyldiethanolamine (MDEA) enabled the separation, in which the tertiary amine enhances the absorption capacity without impacting the EDA's electroactive amine role when mixed in an 80/20 ratio. This formulation reduced charge-transfer resistance by 40% and lowered desorption energetics to 37 $kJ_e/molCO_2$, representing the lowest energy requirement reported for the process [12]. This result demonstrated that efficient EMAR operation depends on the presence of an electroactive amine, and that monoethanolamine

(MEA), although briefly considered as a sorbent in a few studies as a replacement for EDA, cannot fulfill this role because it does not form stable complexes with copper [13, 14]. MDEA is also an industrially important amine for thermal absorption, but it cannot directly replace EDA for the same reason as MEA. This is the basis for retaining EDA in the present work, as it functions both as a fast CO_2 absorbent and as the electroactive ligand that closes the Cu-mediated regeneration cycle. Most recently, the focus has returned to the fundamental reaction kinetics governed by the supporting electrolyte. Investigations revealed that a specific anion effect could facilitate oxidation of the copper surface, which could help in the energetics. However, these newer studies did not account for sulfate, the most commonly used anion in EMAR research, preventing a direct comparison with the majority of existing literature [15].

The thermodynamic insights and cost contributions revealed in early thermodynamic modeling and TEA analysis directly motivated a redesign of the EMAR process. To address the membrane-dominated CapEx, the system was reconfigured into a two-stage, membraneless process. In this design, electrochemical regeneration and gas handling (both absorber and flash tank) are integrated into a compact, stackable cell, eliminating the need for ion-exchange membranes as well as external absorbers, pumps, and flash tanks [16]. Electrode development proceeded in parallel, with attention shifting from high-surface-area metal foams toward gas diffusion electrodes to better control gas transport and maintain access to active copper sites [3, 17].

S2. FUNDAMENTAL ELECTROCHEMICAL CHARACTERIZATIONS

The fundamental electrochemical characterizations were conducted under various conditions. To simulate the flue gas from a natural gas-fired power plant, a mixture containing 5% CO_2, 13% O_2, and 82% N_2 was used. For coal-fired power plants, the simulated flue gas consisted of 13% CO_2, 4% O_2, and 83% N_2. These two cases correspond to the envisioned deployment of small modular electrochemical containers (SMEC) systems for decarbonizing data center electricity supplied by fossil-fuel power plants. To explore broader applicability beyond the data center sector, we also considered a representative scenario for carbon capture from industrial point sources such as cement and steel production, which, together with power plants, constitute the largest stationary contributors to global CO_2 emissions. In this case, a flue gas mixture of 20% CO_2, 4% O_2, and 76% N_2 was employed, reflecting typical average values reported in the literature [18-20]. Finally, two boundary cases, 0% and 100% CO_2, were included to serve as baselines for non-reactive and fully saturated CO_2 environments, respectively. All electrochemical tests in the present work used circular Toray 060 GDEs with a diameter of 4.6 cm. Copper electrodeposition covered approximately 65% of the projected GDE area, while the remaining 35% of the surface remained open for gas exchange. Based on this coverage, the active copper area was 10.80 cm^2 and was used to convert applied current to current density. The electrolyte gap between the two GDEs was 7 mm, and the same gas-channel and electrolyte-cell configuration was used for the natural-gas and coal flue-gas tests.

In our prior work, we finalized the design of the GDEs used in the two-stage process by developing and optimizing an electrochemical deposition method for their fabrication. However, a detailed investigation of the redox behavior of the active species, as well as the associated charge-transfer kinetics and interfacial resistances, remained unaddressed. To fill this gap, we performed cyclic voltammetry (CV) and electrochemical impedance spectroscopy (EIS) under a range of implementation scenarios described earlier, with CO_2 concentrations varying from 0% to 100%.

S2.1. Cyclic Voltammetry

The CV results revealed that both anodic and cathodic currents increased with rising CO_2 concentration in the inlet gas (Figure S2A). This trend can be attributed to enhanced electrolyte conductivity resulting from the higher concentration of dissolved inorganic carbon (DIC), including bicarbonate (HCO_3^-) and carbonate (CO_3^{2-}) species. The increased ionic strength reduces ohmic resistance, enabling higher current densities at a given potential. In addition, a clear anodic peak

shift toward higher potentials was observed with increasing CO_2 content. For example, the anodic peak appeared at −0.4 V for the 0% CO_2 sample and shifted to 0.2 V for the 100% CO_2 condition. This shift can be explained by changes in the equilibrium complexation behavior of Cu^{2+} ions. At 0% CO_2, free EDA is readily available to coordinate with electrochemically generated Cu^{2+}, forming $Cu(EDA)_2^{2+}$ and stabilizing the oxidation state. In contrast, at 100% CO_2, EDA is largely present as EDA–CO_2 adducts, reducing the availability of free EDA. As a result, Cu^{2+} remains uncomplexed in solution for a longer period, requiring a greater driving force, i.e., a higher potential, for oxidation, as dictated by the Nernst equation. Specifically, a higher concentration of the electroactive species, Cu^{2+}, in solution corresponds to a higher Nernstian potential (*E*): $E = E^0 + \frac{RT}{2F}\ln[Cu^{2+}]$. This shift reflects the thermodynamic penalty associated with complexation in a CO_2-saturated medium.

Samples with higher CO_2 concentrations (notably 20% and 100%) also exhibited two distinct anodic peaks. The first peak, at a lower potential, corresponds to the initial electron transfer step ($Cu^0 \rightarrow Cu^+ + e^-$), while the second peak represents the subsequent oxidation ($Cu^+ \rightarrow Cu^{2+} + e^-$). The separation of these peaks became more apparent at high CO_2 concentrations due to delayed and relatively less efficient coordination of Cu^+ and Cu^{2+} with EDA, again arising from the limited availability of free EDA. At lower CO_2 levels, where EDA is more freely available, both oxidation steps occur rapidly and in close succession, resulting in a merged single anodic peak. Similar anodic peak shifts and separations have been previously observed in the four-stage EMAR system under variations in electrolyte composition, additive concentrations, and operational conditions, further validating the role of complexation kinetics and thermodynamics in modulating electrochemical behavior.

For the cathodic peaks, only a single reduction peak was observed across all gas compositions, with no measurable shift in peak potential. This behavior is expected, as CO_2 is neither directly nor indirectly involved in the reduction reaction: $Cu(EDA)_2^{2+} + 2e^- \rightarrow Cu^0 + 2EDA$. In this step, the complexed Cu^{2+} species is reduced to metallic copper, and EDA is released; the presence of CO_2 does not significantly alter the redox potential of this process. However, consistent with the anodic behavior, the cathodic peak current increased with rising CO_2 concentration. This trend is attributed to the increased concentration of DIC, which enhances electrolyte conductivity and reduces ohmic resistance, thereby enabling higher current densities.

Overall, the CV analysis highlights the strong coupling between gas composition and electrochemical behavior in the two-stage EMAR system. Increasing CO_2 concentration impacts

both electrolyte conductivity and the coordination environment of redox-active species, leading to notable shifts in anodic peak potentials and peak separations. In contrast, the cathodic process remains unaffected in potential but exhibits enhanced current due to reduced ohmic losses. These insights confirm the redox activity and reversibility of the Cu–EDA–CO_2 cycle under practical flue gas conditions and provide a mechanistic foundation for tuning EMAR performance across a range of CO_2 capture scenarios.

S2.2. Electrochemical Impedance Spectroscopy

EIS data were collected under all CO_2 concentrations. The corresponding Nyquist plots (Figure S2B) display single, well-defined semicircles with no discernible Warburg tail, indicating that charge-transfer kinetics dominate interfacial impedance and mass-transport limitations are negligible under these conditions. The solution resistance (R_s), as given by the high-frequency intercept on the real axis, remains essentially constant at 5-10 Ω. In contrast, the diameter of the semicircle, i.e., the charge-transfer resistance (R_{ct}), increases proportionally with CO_2 concentration, confirming symmetric kinetic barriers for the redox reaction. This is attributed to the progressive sequestration of free EDA into EDA–CO_2 adducts, thereby reducing the availability of unbound ligand for efficient Cu^{2+}/Cu^0 coordination. In both the reduction half-reaction and the corresponding oxidation process, this reduction in ligand accessibility results in slower complexation kinetics, which manifest as increased interfacial resistance. These impedance results are consistent with the CV findings. While elevated CO_2 concentrations improve bulk conductivity and thus enable higher peak currents in CV, the concomitant rise in R_{ct} introduces larger overpotentials, reflected in the anodic peak shifts. From a process design perspective, these findings emphasize the importance of maintaining adequate free EDA concentrations at the electrode interface. Achieving an optimal balance between high CO_2 loading for process throughput and low interfacial resistance for kinetic efficiency will be critical to system performance.

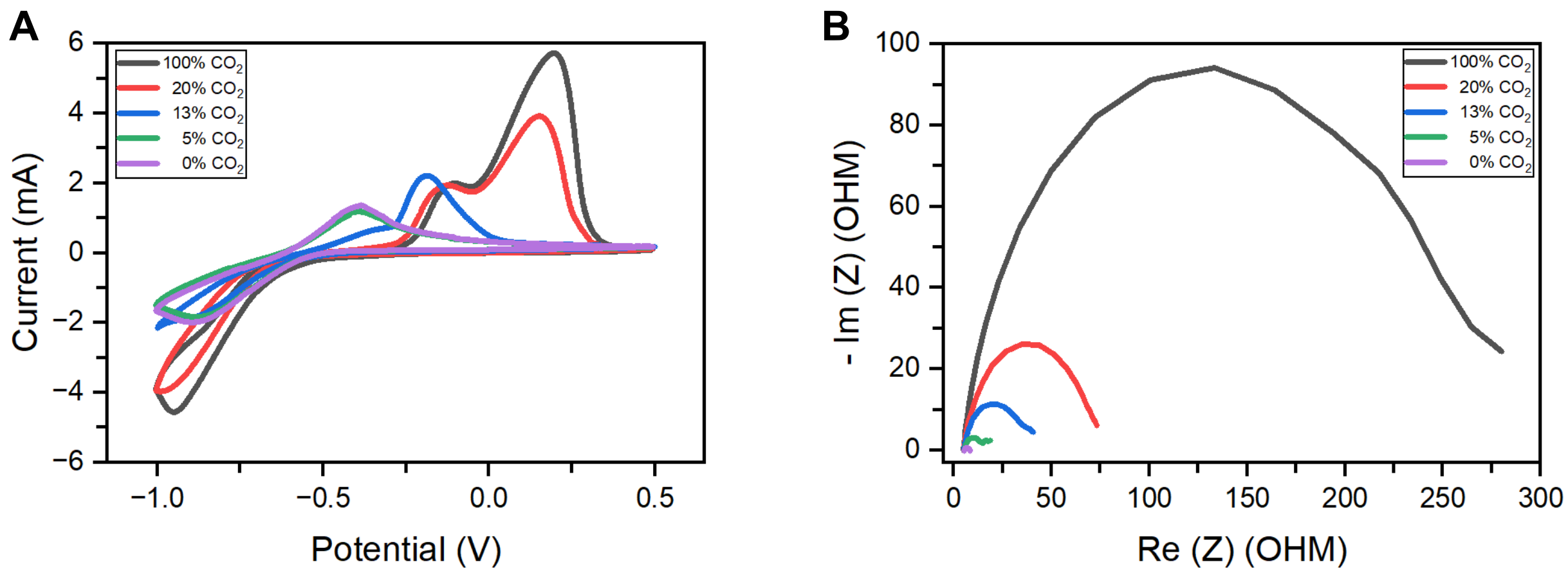

Figure S2. Fundamental electrochemical characterization across CO_2 concentrations. (A) Cyclic voltammetry (CV) and (B) electrochemical impedance spectroscopy (EIS) responses of the two-stage membraneless EMAR system measured under gas mixtures containing 0%, 5%, 13%, 20%, and 100% CO_2. The 5% and 13% CO_2 conditions represent natural-gas and coal-derived flue gases, respectively; 20% CO_2 corresponds to a typical industrial point-source stream (e.g., cement or steel), while 0% and 100% CO_2 serve as non-reactive and fully CO_2-saturated baselines, respectively.

S3. ELECTRODE & ELECTROLYTE STABILITY AFTER LONG-TERM CYCLING

This section provides a comprehensive post-cycling diagnostic analysis of both electrodes and electrolyte following 300 polarity-switching cycles. Both morphological and compositional measurements, including scanning electron microscopy (SEM), titration, gas chromatography (GC), and ion chromatography (IC) analyses, were employed to identify any traces of degradation and to verify the long-term chemical and electrochemical stability of the two-stage EMAR system.

S3.1. Morphological Characterization of GDEs After 300 Cycles

GDEs were examined to evaluate whether extended cycling introduced morphological degradation that could compromise electrochemical performance. SEM was first conducted on a freshly electrodeposited GDE used as both the anode and cathode (Figure S3A). This electrode exhibited a uniform copper loading of 1.7 mg/cm^2 deposited onto the carbon fiber network, providing a consistent baseline for comparison. Figures S3B and S3C show the GDE after serving as the cathode and anode, respectively, during the final cycle of long-term operation under the natural gas scenario, while Figures S3D and S3E correspond to the coal-based scenario. During the 300-cycle experiment, electrode polarity was periodically switched, resulting in copper corrosion at the anode and deposition at the cathode in each cycle. As expected, the GDE operating as the cathode in the final cycle exhibited a higher copper loading than the fresh electrode. In contrast, under the coal-based scenario, which operated at higher current densities, a larger partial imbalance between the anode and cathode relative to the fresh GDE was observed. For both samples, the overall structural integrity of the GDEs was preserved across all tested conditions.

Across all samples, the carbon-based gas diffusion layer (GDL) retained its structural integrity, with no evidence of pore collapse, delamination, or binder failure. The mesoporous scaffold also remained intact, maintaining the open pore network required for gas transport. The electrodeposited copper domains displayed the expected granular morphology characteristic of Cu^0 cycling through repeated oxidation and reduction steps; however, no abnormal particle growth, dendritic protrusions, or dense passivation films were observed. The copper features remained uniformly distributed, and their size and texture were comparable to those observed before cycling. No cracking, tearing, or mechanical abrasion of the GDE surfaces was detected, indicating that the periodic polarity switching, implemented specifically to avoid unidirectional copper accumulation, successfully mitigated asymmetric metal buildup or localized degradation.

Additionally, no evidence of irreversible surface passivation (e.g., sulfate- or nitrate-derived films, carbonate crusts, or amine-derived polymeric deposits) was observed, consistent with the stable electrochemical behavior and near-unity voltage-area ratios reported in the main text. Overall, the SEM analysis confirms that the GDEs exhibit excellent morphological stability under accelerated cycling conditions. The preservation of scaffold structure, integrity of the GDL, and reversible copper morphology collectively indicate that the electrode architecture is well-suited for prolonged operation within the two-stage EMAR process and provides strong justification for scaling to stacked and modular SMEC configurations.

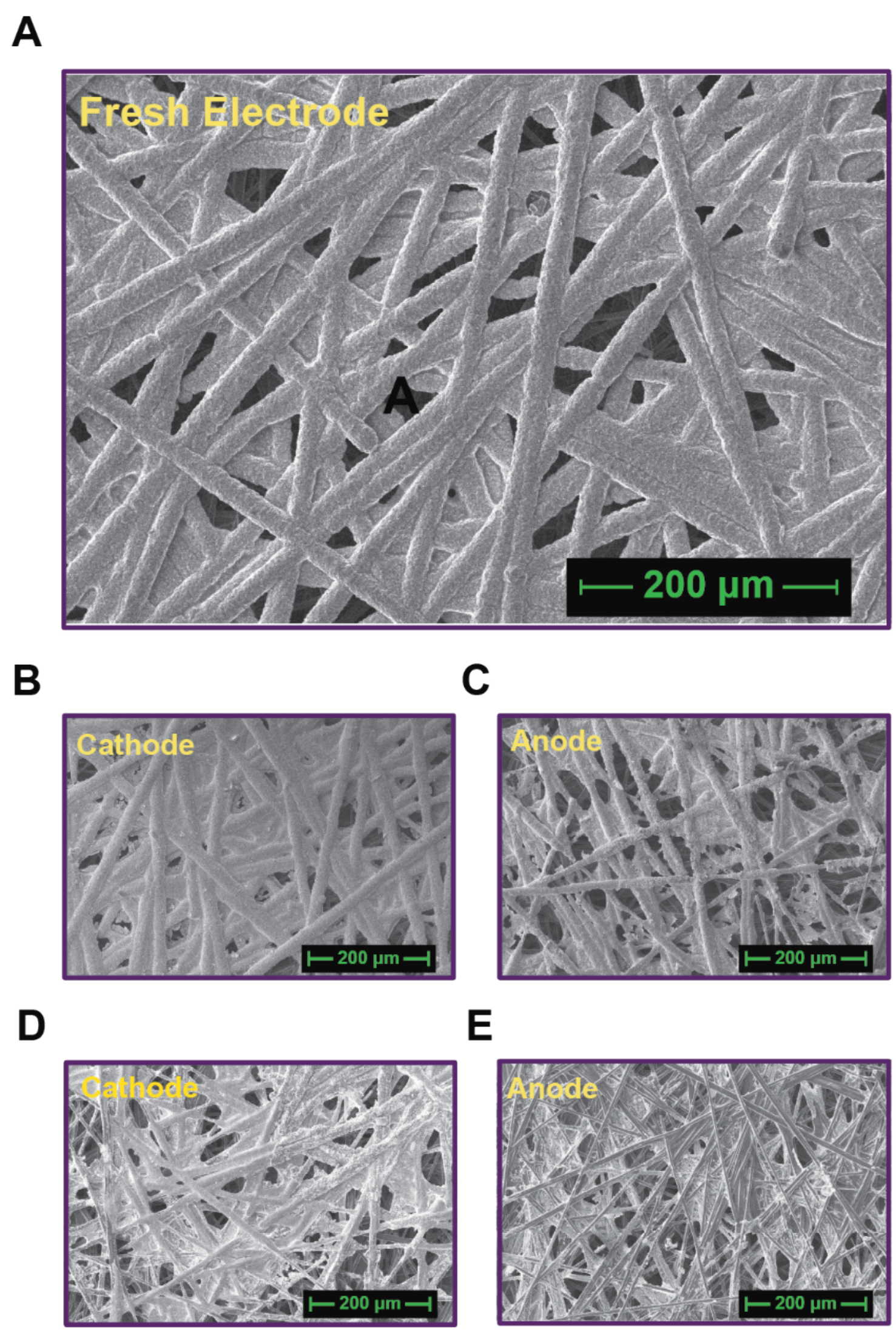

Figure S3. Morphological evolution of GDEs during long-term cycling. Scanning electron microscopy (SEM) images of (A) a freshly electrodeposited GDE with a uniform copper loading of 1.7 mg/cm^2 on a carbon fiber network; (B) the GDE after serving as the cathode and (C) as the anode in the final cycle of long-term operation under the natural-gas scenario; and (D) the cathode and (E) the anode after the final cycle under the coal-based scenario.

S3.2. Electrolyte Composition and Amine Stability Analysis

The chemical stability of the electrolyte and the integrity of the EDA absorbent were assessed following 300 polarity-switching cycles. Total EDA concentration was quantified by acid–base titration before and after cycling. The measured EDA concentrations were indistinguishable within experimental uncertainty, indicating that no measurable thermal, oxidative, or redox-induced degradation of the amine occurred during long-term operation. This study did not directly quantify EDA volatilization or long-duration operation beyond 50 h. Therefore, future validation should include controlled operation under both inert flue gas streams and oxygen-containing flue gases to quantify the impact of oxygen on the amine. It's important because practical SMEC operation would require closed electrolyte handling, leak monitoring, solvent makeup control, and proper recycling of copper-containing electrolyte. To further evaluate absorbent integrity, the maximum CO_2 loading capacity of the electrolyte was determined prior to and following the 300-cycle test. Identical CO_2 loadings were obtained in both measurements, demonstrating that the carbamate formation capacity of EDA was fully preserved. Because many known EDA degradation pathways produce functional groups (e.g., aldehydes, amides, oligomers) that reduce the number of available amine sites [21-23], any meaningful molecular degradation would have manifested as a decrease in CO_2 loading; none was observed.

GC was used to continuously analyze the gas exiting the gas channels on the working electrode (WE) side over 10 consecutive cycles (100 min total) under operating conditions identical to those used in the long-term experiments for the natural gas and coal-based scenarios. The same inlet gas configuration was applied to both the anode and cathode in each scenario to enable direct determination of the percentage of CO_2 removed, which was only possible in the presence of N_2 and O_2 in the sweep-gas stream. In the final operating configuration, the anode side should receive a sweep gas while the cathode side receives flue gas, and these streams should be switched synchronously with polarity reversal. This synchronized gas-switching arrangement was not available in the present setup. Using a pure CO_2 sweep would also mask the change in CO_2 concentration measured by GC and could increase the CO_2 partial pressure at the anode, while a water-vapor sweep would require condensation before CO_2 compression. The present GC experiment was therefore designed to quantify reversible single-pass CO_2 capture and desorption under controlled $N_2/O_2/CO_2$ mixtures, not to optimize product purity. Each cycle consisted of an initial open-circuit voltage (OCV) with no applied current, followed by either anodic polarization for CO_2 desorption or cathodic polarization for CO_2 absorption, with each step maintained for 5 min before switching.

Figure S4A shows the GC peaks of CO_2 at three characteristic points in the cycling sequence: OCV at time zero, anodic desorption at 50 min, and cathodic absorption at 100 min. As expected, the coal-based scenario consistently exhibits higher CO_2 peaks due to its higher inlet CO_2 concentration (13%) compared to the natural gas case (5%). The inset in Figure S4A shows the corresponding N_2 and O_2 peaks detected on GC Channel A, demonstrating that their concentrations remain unchanged during both absorption and desorption steps. This confirms selective electrochemical absorption of CO_2, with no detectable crossover of N_2 or O_2 across the GDE interface. Throughout the experiment, only CO_2, N_2, and O_2 were detected, with no evidence of ammonia or other nitrogen-containing volatile degradation products, confirming the absence of amine degradation under the operating conditions. GC did not detect H_2, CO, CH_4, NH_3, or other gaseous products, supporting the absence of measurable hydrogen evolution reaction (HER) or CO_2 reduction (CO2RR) under the tested conditions. Figure S4B shows the variation in CO_2 concentration (%) over the full 100 min experiment. The area under the CO_2 peak is proportional to the CO_2 concentration in the sample, which was calculated by integrating the peak area in the retention time range (0.325-0.350 retention time) for each sample. Each data point corresponds to an individual GC sampling and analysis, with a minimum acquisition time of 1.5 min per measurement. For the natural gas scenario, the outlet CO_2 concentration variation was between ~13.5% during anodic desorption and ~2% during cathodic absorption, while for the coal-based scenario it ranged from approximately 22% to 5%. GC analysis of the gas stream at anodic cycle likewise revealed that the desorbed product consisted exclusively of CO_2, confirming the absence of parasitic electrochemical pathways capable of generating gaseous byproducts. Together, titration, CO_2-loading measurements, and GC analysis demonstrate that the electrolyte composition remains unchanged, and the EDA absorbent maintains full chemical integrity over extended cycling. These findings corroborate the electrochemical stability observed in the long-term voltage-area analysis.

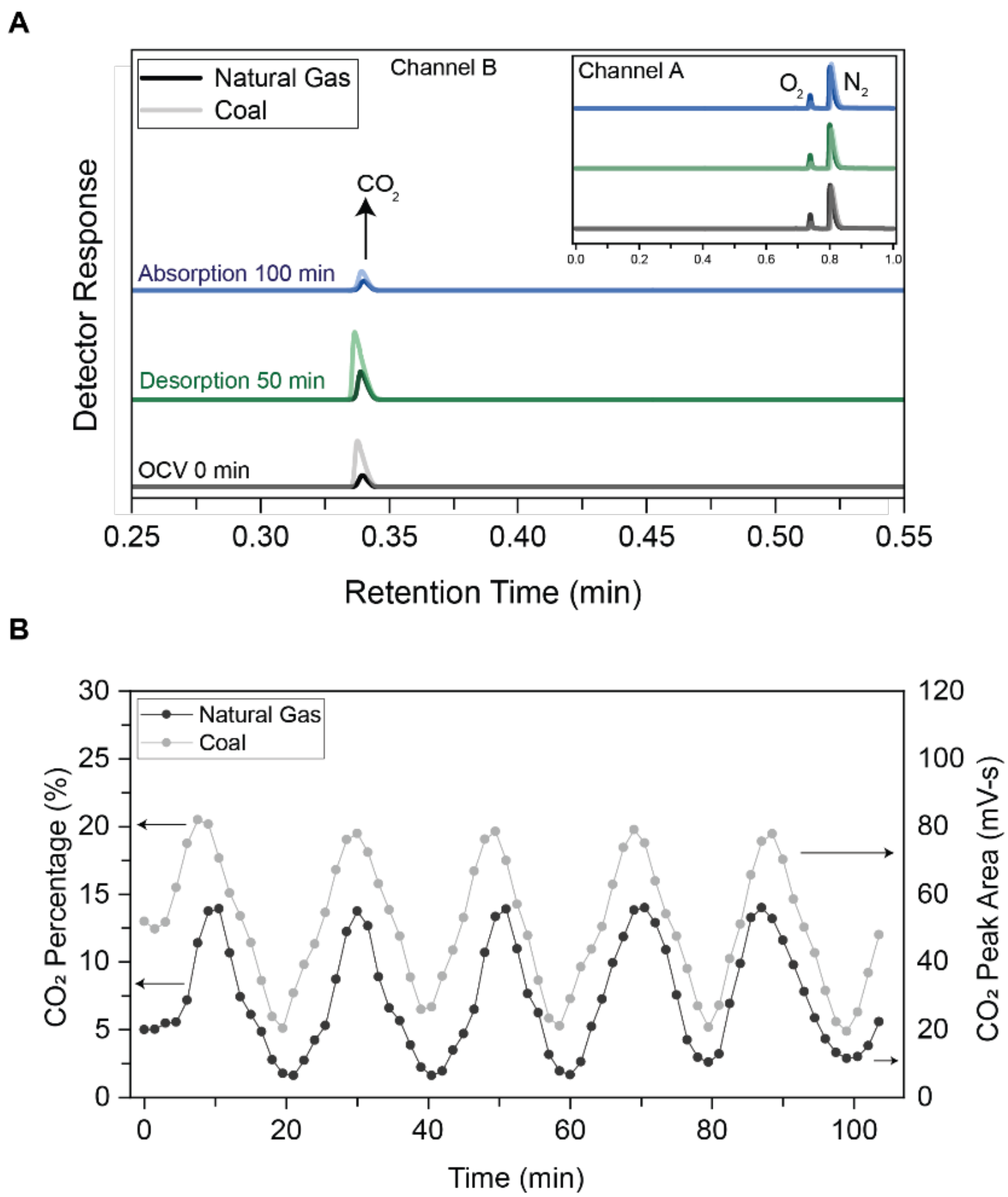

Figure S4. Online Gas chromatography (GC) analysis of CO_2 absorption and desorption. GC was conducted on the gas exiting the working-electrode gas channels over 10 cycles (100 min) under operating conditions identical to the long-term experiments for the natural gas (5% CO_2) and coal-based (13% CO_2) scenarios. (A) CO_2 peaks at OCV (0 min), anodic desorption (50 min), and cathodic absorption (100 min); N_2 and O_2 peaks (inset), confirming selective CO_2 absorption with no gas crossover or detectable degradation products. (B) CO_2 concentration (%), calculated from the integrated peak area from the retention time (0.325 to 0.350 min channel B) at every sampling interval spanning 1.5 min.

S3.3. Trace Species Detection via IC and UV-Vis

IC was employed to quantify trace anionic species in the electrolyte following the operation. Across all samples, sulfate (SO_4^{2-}), introduced exclusively from the supporting electrolyte, was the only anion detected. The combined IC and GC results support the carbon-balance assessment, because no products were detected that relate to CO_2 reduction or solvent oxidation.

Figure S5 represents the IC spectra for fresh electrolyte and both the natural gas and the coal-based electrolytes after the long-term experiment. The regions highlighted show the peak locations for the respective anions. The concentration remained unchanged relative to the pre-operation baseline, confirming that no oxidative or reductive decomposition pathways produced new anionic species such as formate, acetate, oxalate, nitrite, or nitrate, which are commonly formed during amine degradation or parasitic solvent oxidation in other CO_2 capture systems. The absence of these degradation fingerprints further supports the conclusion that EDA remains chemically intact and does not undergo degradation under the cycling conditions. The IC results also reflect the chemical stability of the GDEs. Degradation of the GDE components, such as oxidation of the carbon support or hydrolysis of fluorinated binders, would be expected to introduce characteristic anionic species into the electrolyte (e.g., fluoride from PTFE/PTFE-like binders, formate or acetate from carbon oxidation, or trace carboxylates from polymer side-chain breakdown). The complete absence of these species in the IC chromatograms indicates that no detectable GDE-derived anions were released during long-term operation, further supporting the structural and chemical robustness of the electrode architecture. These IC results also support the carbon-balance assessment, because no nitrate and nitrite were detected as possible products of CO_2 reduction. It also confirms that there was no evidence of possible oxidative amine degradation in the oxygen-containing flue-gas tests.

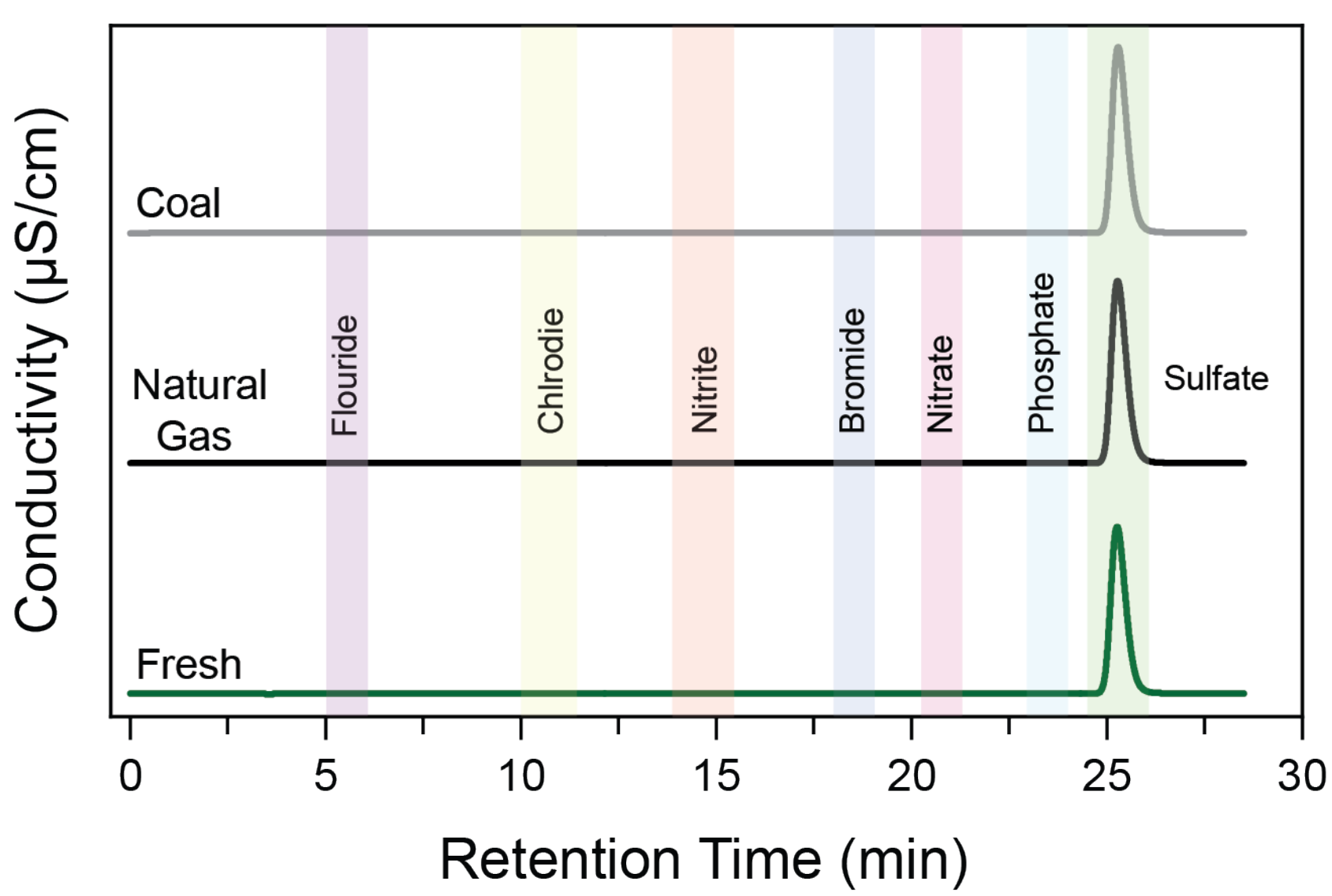

Figure S5. Ion chromatography (IC) analysis of electrolyte anionic composition. IC spectra are shown for the fresh electrolyte and for electrolytes recovered following long-term continuous operation under natural gas (5% CO_2) and coal-based (13% CO_2) flue gas conditions. Sulfate (SO_4^{2-}), originating from the supporting electrolyte, was the only anion detected in all samples, with no change in concentration relative to the pre-operation baseline. The expected retention times for fluoride, chloride, nitrite, bromide, nitrate, phosphate, and sulfate are highlighted by shaded regions on the chromatograms, indicating the time windows at which these anions appear in the IC spectra.

Copper concentration in the electrolyte was quantified using UV–Vis spectroscopy (Figure S6). A calibration curve was first established using standard solutions with copper concentrations ranging from 0.1 to 0.5 M before analyzing the electrolytes collected at the end of the long-term experiments for both the natural gas and coal-based scenarios. The calibration curve shows a linear increase in absorbance with increasing copper concentration, which is also visually evident from the progressive darkening of the electrolyte color. A calibration standard of 0.25 M copper was used as the reference point, corresponding to the initial copper concentration used in the experiments. Analysis of the long-term electrolyte sample for the coal combustion scenario indicated higher apparent copper concentrations. This minor loss of the copper electrode to the electrolyte, likely due to the high gas evolution during the desorption step, can be effectively eliminated by using additives (e.g., surfactant), as fully investigated before [9]. The periodic polarity-switching protocol was used specifically to avoid one-directional copper depletion at one GDE and copper buildup at the other, and these results do not indicate excessive copper imbalance over the accelerated test.

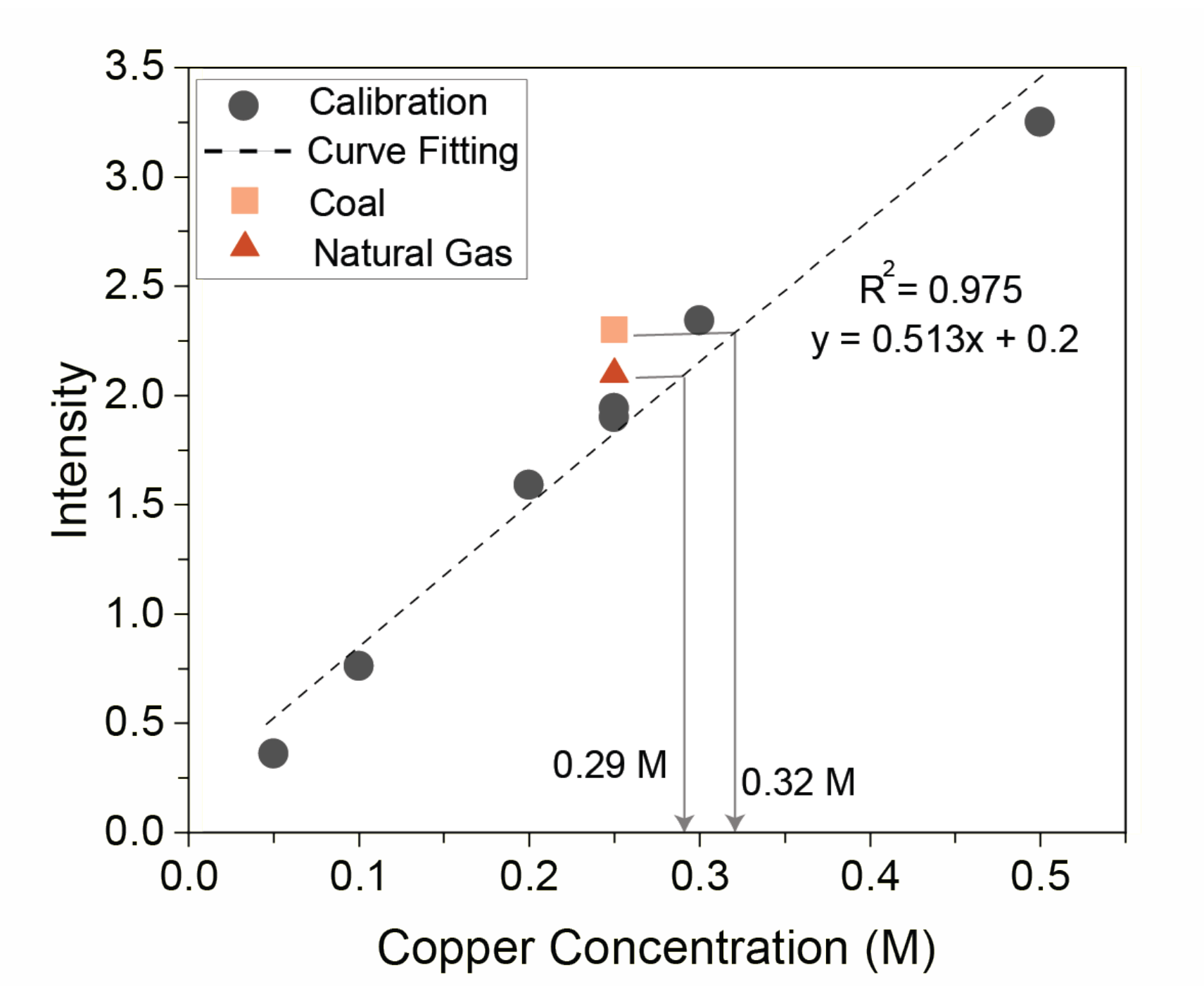

Figure S6. UV–Vis analysis of copper concentration in the electrolyte. A calibration curve was generated using copper standards (0.1–0.5 M), with 0.25 M serving as the reference corresponding to the initial experimental concentration. Electrolyte samples collected after long-term operation under natural gas and coal-based conditions were diluted 1:7 (v/v) before analysis to maintain absorbance within the linear range of the instrument.

S4. COMPARISON OF ENERGETICS WITH OTHER ECCs

Recent electrochemical carbon capture (ECC) literature is broad in mechanism and uneven in reporting basis. For the present manuscript, the most relevant comparison is not to the entire ECC field, but to the smaller subset of studies that (i) treat a gas-phase CO2 feed, (ii) operate at point-source-relevant concentrations, and (iii) report a measured electrical capture/release metric on a $kJ_e/molCO_2$ basis. Within the recent gas-fed, post-combustion-relevant set, several high-impact technologies provide useful external context. A modular porous-solid-electrolyte (PSE) reactor operated with gas feeds containing 10.7%, 5.6%, 4.1%, and 1.2% CO_2 was reported, and described full-cell carbon-capture energy beginning near 90 $kJ_e/molCO_2$ [24]. In another study, a decoupled redox system on a simulated coal flue gas containing 15% CO_2 and 5% O_2 was demonstrated, reporting 49.16 $kJ_e/molCO_2$ at 10 mA/cm^2 and 92.93 $kJ_e/molCO_2$ in a scaled system producing about 0.4 kg CO_2 daily [25]. Jin et al. [26] reported 73 $kJ_e/molCO_2$ for simulated flue gas and 104 $kJ_e/molCO_2$ for DAC in a hybrid phenazine flow cell, while Zhu et al. [27] reported continuous capture with energy beginning around 150 $kJ_e/molCO_2$ and production of >99% CO_2. Jin et al. [28] further provided a transparent pressure-based pH-swing benchmark of 61.3 $kJ_e/molCO_2$ for separation from 0.1 bar CO_2 to pure CO_2 at 1 bar. Pang et al. [29] reported a lower 36-55 $kJ_e/molCO_2$ range, but that result used 10% CO_2 capture with a pure-CO_2 sweep for release, which reflects a more favorable release boundary than a strict post-combustion separation process.

The broader ECC field remains important for context, but many papers are only indirectly comparable to the present study. Recent molecular and redox-carrier papers include the redox-tunable Lewis-base study, which cycled under 15% CO_2 with 5% O_2 [30], and a system with benzodithiophene quinone (BDT-Q) as the redox molecule, which operated for more than 100 h in a simulated flue gas containing 13% CO_2 and 3.5% O_2 [31]. Another system with quinone as the redox-active molecule also demonstrated continuous aqueous capture/release under 15% CO_2 and 3% O_2 [32]. These studies are useful because they establish the importance of O_2 tolerance and molecular stability, but they do not provide the same level of directly comparable full-cycle energy reporting as the previously mentioned systems.

Several adjacent ECC technologies should also be treated carefully because their process boundary is fundamentally different from gas-phase post-combustion capture. Zhang et al. [33] reported 50-118 $kJ_e/molCO_2$ for electrochemical regeneration from carbonate/bicarbonate solutions in a porous solid-electrolyte reactor, and Bui et al. [34] analyzed bipolar-membrane systems for DAC and oceanwater capture with modeled DAC energies below 150 $kJ_e/molCO_2$.

These systems are therefore informative as adjacent ECC, especially when discussing electrochemical alkalinity generation, but they should not be used as direct energy benchmarks for a gas-fed EMAR process.

Within the EMAR family itself, prior post-combustion studies remain important background but should be framed as same-family context. Stern et al. [1] established the foundational EMAR concept and reported bench-scale work below 100 $kJ_e/molCO_2$. Wang et al. [6] then demonstrated >80% separation from a 15% CO_2 feed at 40-80 $kJ_e/molCO_2$, and Rahimi et al. [5] reported 100 h stable operation with a mixed absorbent. The prior membraneless EMAR paper further established the two-stage architecture at the chemistry and cell level, with >90% removal efficiency and energy as low as 60 $kJ_e/molCO_2$ [16]. A full comparison of the electrical energetics of the developed process with those reported for other ECC systems is provided in Figure S7.

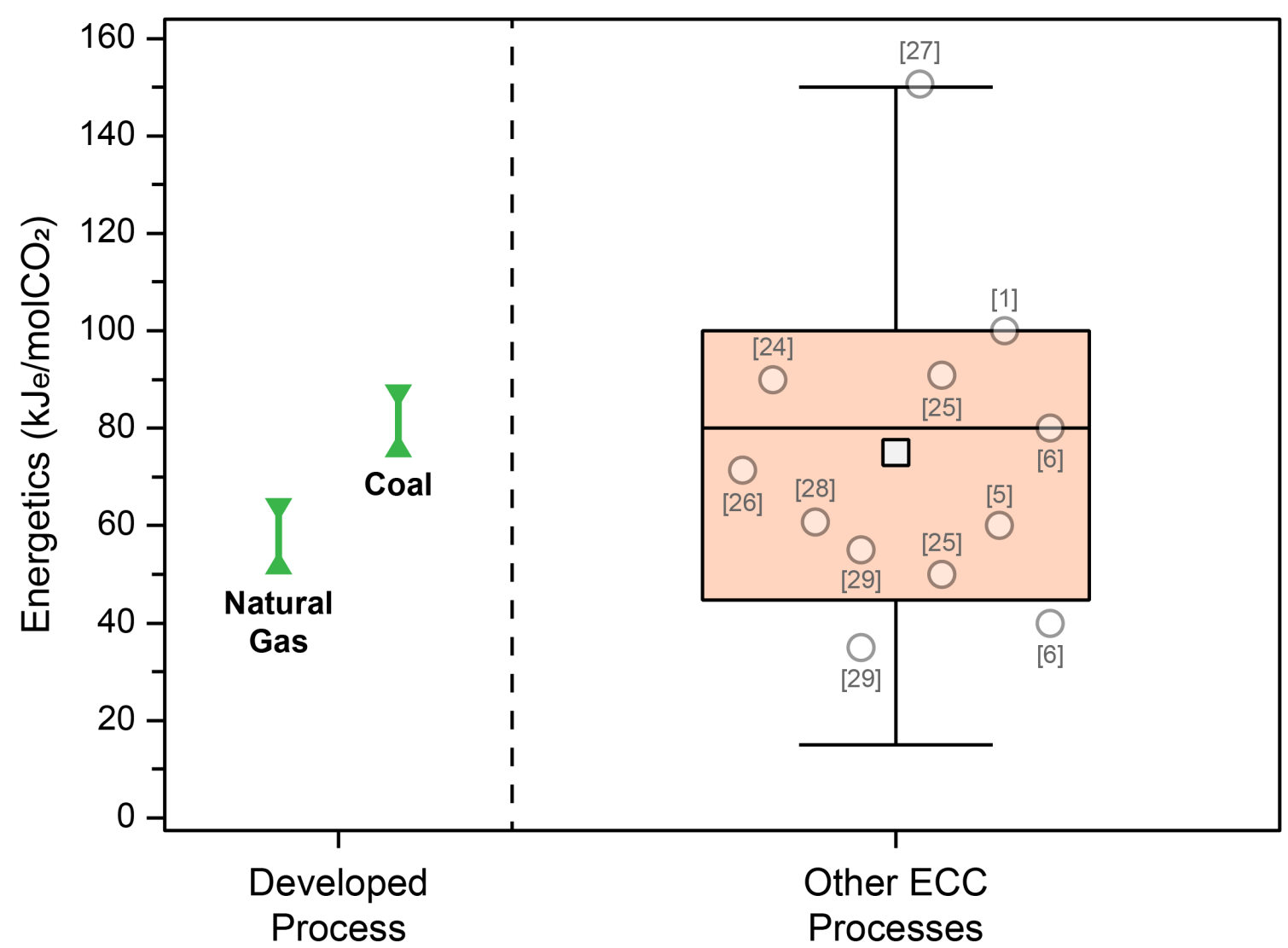

Figure S7. Electrical energetics of the developed membraneless EMAR process compared with other reported ECC systems. (Left) Experimentally observed range of electrical energetics for the developed process under natural-gas-like and coal-like flue gas conditions. **(Right)** Literature distribution for other gas-fed or post-combustion-relevant ECC systems reporting measured energy metrics on a $kJ_e/molCO_2$ basis. Open circles represent some examples of recent reports, and bracketed numbers denote the corresponding references. The box, center line, square, and whiskers represent the interquartile range, median, mean, and full range, respectively. Differences in process boundaries and operating conditions across studies should be considered when interpreting direct comparability.

S5. MODELING FRAMEWORK FOR SO_2 & NO IMPURITY EFFECTS

This section details the modeling tools used to quantify the chemical and electrochemical impacts of SO_2 and NO impurities on the two-stage EMAR process. Because handling these impurities experimentally poses safety and regulatory challenges, their effects were evaluated through rigorously formulated thermodynamic and kinetic models. The results are therefore used as an impurity-risk assessment and do not represent direct experimental validation of SO_2 or NO tolerance. The SO_2 framework captures all relevant homogeneous equilibria governing electrolyte speciation, while the NO framework incorporates competitive electrode kinetics, transport limitations, and Faradaic efficiency analysis.

S5.1. Thermodynamic Modeling Framework for SO_2

A comprehensive thermodynamic speciation model was developed to quantify the influence of SO_2 impurities on the chemical environment of the Cu–EDA electrolyte used in the two-stage EMAR process under natural-gas and coal flue gas scenarios. The model accounts for gas dissolution, acid-base chemistry, amine protonation, metal-ligand complexation, carbonate and sulfite equilibria, and charge/mass balances, enabling quantitative prediction of pH, EDA speciation, Cu-complex concentrations, and total CO_2 absorption capacity. All equations, equilibrium constants, and model assumptions are provided such that the full calculation can be reproduced using any numerical solver.

System Description and Assumptions

The model considers dissolved CO_2 and SO_2 arising from gas streams representative of: (i) Natural gas flue gas: 5% CO_2, trace SO_2 (0.01-10 ppb); (ii) Coal flue gas: 13% CO_2, elevated SO_2 (1 ppb-2000 ppm). For sensitivity analyses, SO_2 was varied over several orders of magnitude to simulate flue gas with and without pretreatment. Dissolved gas concentrations were computed as:

$$[H_2CO_3] = H_{CO2} \times P_{CO2} \qquad H_{CO2} = 0.034 \text{ M/atm} \qquad \text{(S1)}$$

$$[H_2SO_3] = H_{SO2} \times P_{SO2} \qquad H_{SO2} = 1.230 \text{ M/atm} \qquad \text{(S2)}$$

Chemical Equilibria Included in the Model

All reactions and equilibrium constants (K) are listed. For clarity, reactions are grouped into several categories:

Table S1. Equilibrium reactions and corresponding K values included in the chemical speciation model

Category	Reaction	$\log_{10} K$	Equation
Water Autoionization	$H^+ + OH^- \rightleftharpoons H_2O$	−13.997	(S3)
Carbonate System	$H_2CO_3 \rightleftharpoons HCO_2^- + H^+$	−6.35	(S4)
	$HCO_3^- \rightleftharpoons CO_3^{2-} + H^+$	−10.329	(S5)
Sulfite System	$H_2SO_3 \rightleftharpoons HSO_3^- + H^+$	−1.85	(S6)
	$HSO_3^- \rightleftharpoons SO_3^{2-} + H^+$	−7.19	(S7)
Amine Protonation and Carbamate Formation	$EDA + H^+ \rightleftharpoons EDAH^+$	9.928	(S8)
	$EDAH^+ + H^+ \rightleftharpoons EDAH_2^{2+}$	6.848	(S9)
	$EDA + CO_{2(aq)} \rightleftharpoons EDACO_2$	5.826	(S10)
Copper System	$Cu^{2+} + 2\ EDA \rightleftharpoons Cu(EDA)_2^{2+}$	19.58	(S11)
	$Cu^{2+} + OH^- \rightleftharpoons CuOH^+$	−7.497	(S12)
	$Cu^{2+} + CO_3^{2-} \rightleftharpoons CuCO_3$	6.77	(S13)
	$Cu^{2+} + HCO_3^- + H^+ \rightleftharpoons CuHCO_3^+$	12.129	(S14)
	$Cu^{2+} + SO_4^{2-} \rightleftharpoons CuSO_4$	2.36	(S15)
	$Cu^{2+} + HSO_4^- \rightleftharpoons CuHSO_4^+$	2.34	(S16)
	$Cu^{2+} + SO_3^{2-} \rightleftharpoons CuSO_3$	5.39	(S17)
Sodium System	$Na^+ + HCO_3^- + H^+ \rightleftharpoons NaHCO_3$	10.029	(S18)
	$Na^+ + CO_3^{2-} \rightleftharpoons NaCO_3^-$	1.27	(S19)
	$Na^+ + SO_4^{2-} \rightleftharpoons NaSO_4^-$	0.74	(S20)
	$Na^+ + SO_3^{2-} \rightleftharpoons NaSO_3^-$	0.83	(S21)
Sulfate System	$HSO_4^- \rightleftharpoons SO_4^{2-} + H^+$	−1.99	(S22)

Mass Balance and Charge Balance Equations

The following total species balances were enforced: sodium balance, EDA balance (including consumption by Cu–EDA formation), carbon balance (including DIC + carbamate + metal carbonates), sulfite/SO_2 balance, sulfate balance, and copper balance.

Charge neutrality was enforced using: ∑cations = ∑anions, where:

Cations: H^+, $EDAH^+$, $EDAH_2^{2+}$, Cu^{2+}, Cu complexes, Na^+

Anions: OH^-, HCO_3^-, CO_3^{2-}, HSO_3^-, SO_3^{2-}, SO_4^{2-}, etc.

Numerical Solution Strategy

The system (28 nonlinear equations) was solved using Python programming language with: SciPy *fsolve*, tolerance 1×10^{-16}; Initial guess scaling strategy: multiplicative adjustment of the initial guess if error > 1×10^{-12}; Solver iterated automatically until all equations satisfied. A full set of 28 unknowns is solved simultaneously.

Parameter Sets for Different Flue-Gas Scenarios

To reproduce Figures 6A and 6B in the main text, the following two scenarios were considered:

Table S2. Flue gas compositions and model input parameters for natural gas and coal scenarios

Scenario	P_{CO2} [atm]	P_{SO2} [atm]
Natural Gas Power Plant	0.05	1×10^{-11} to 1×10^{-8} [a]
Coal Power Plant	0.13	1×10^{-9} to 2×10^{-3} [b]

[a] *corresponding to 0.01-10 ppb*
[b] *corresponding to 1 ppb-2000 ppm*

Other parameters for both scenarios were [$CuSO_4$] = 0.1 M; [Na_2SO_4] = 0.5 M; [EDA] = 1.0 M. From the converged solution, the following were computed: pH = $-\log_{10}$ [H^+]; Total CO_2 absorbed (DIC + carbamate + metal carbonates); EDA speciation (free EDA, $EDAH^+$, $EDAH_2^{2+}$, carbamate, Cu–EDA-bound EDA); Cu complex distribution; Degree of EDA protonation → direct indicator of SO_2-induced capacity loss; Fraction of EDA unavailable for CO_2 capture. Finally, these values were used to construct Figure 6.

S5.2. Kinetic model for NO reduction and Faradaic efficiency analysis

In contrast to SO_2, nitric oxide (NO) does not participate in any appreciable homogeneous acid-base or complexation chemistry. NO was therefore treated purely as an electroactive impurity that can compete with the desired $Cu(EDA)_2^{2+}$ reduction and hydrogen evolution at the cathode. The objective of this analysis was to quantify the upper bound on the Faradaic efficiency (FE) loss that could be attributed to NO reduction over the range of concentrations relevant to natural gas and coal flue gases.

Reaction Set

The cathode was modeled as hosting the following five parallel half-reactions:

Copper deposition: $$Cu(EDA)_2^{2+} + 2e^- \rightarrow Cu^0 + 2EDA \quad (S23)$$

Hydrogen evolution reaction:	$H^+ + e^- \rightarrow H_2$	(S24)
NO reduction to nitrous oxide:	$2NO + 2H^+ + 2e^- \rightarrow N_2O + H_2O$	(S25)
NO reduction to nitrogen:	$2NO + 4H^+ + 4e^- \rightarrow N_2 + 2H_2O$	(S26)
NO reduction to ammonia:	$NO + 5H^+ + 5e^- \rightarrow NH_3 + H_2O$	(S27)

These three NO pathways (Equations S25-S27) span the major experimentally observed products of aqueous NO electroreduction (N_2O, N_2, and NH_3) on metal electrodes under mildly acidic/neutral conditions and collectively capture 2-5 electron transfer stoichiometries. Routes that proceed via nitrite or nitrate intermediates require O-atom transfer chemistry and are generally associated with oxidative or disproportionation processes; they are therefore expected to be minor under the strongly cathodic, gas-diffusion conditions considered here and were neglected. Excluding these additional channels is conservative, because any extra NO reduction pathways would further dilute the NO-related FE and thus reduce the apparent impact of NO on the overall cathodic current.

Butler-Volmer Kinetics for Parallel Reactions

Each half-reaction i was described by a Butler-Volmer expression for the local current density j_i (A m^{-2}):

$$j_i \;=\; j_{0,i}\left[\exp\left(\frac{\alpha_i n_i F}{RT}(E - E_{\text{eq},i})\right) - \exp\left(-\frac{(1-\alpha_i) n_i F}{RT}(E - E_{\text{eq},i})\right)\right] \qquad \text{(S28)}$$

where, $j_{0,i}$is the exchange current density for reaction i (A m^{-2}), α_i is the transfer coefficient (–), n_i is the number of electrons transferred in reaction i (–): 2 for Equations S23-S25, 4 for Equation S26, and 5 for Equation S27, F is Faraday's constant (96,485 C mol^{-1}), R is the molar gas constant (8.314 J mol^{-1} K^{-1}), T is temperature (K; taken as 298 K), E is the local electrode potential (V vs. SHE), and $E_{\text{eq},i}$ is the formal equilibrium potential (V vs. SHE) for reaction i under the local activities of reactants and products.

The equilibrium potentials were computed from the Nernst equation:

$$E_{\text{eq},i} \;=\; E_i^\circ \;+\; \frac{RT}{n_i F}\ln Q_i \qquad \text{(S29)}$$

where E_i° is the standard reduction potential (V vs. SHE) and Q_i is the reaction quotient written in terms of activities of the species appearing in Equations S23-S27. Standard potentials for the Cu^{2+}/Cu and H^+/H_2 couples were taken from standard electrochemical tables (e.g., Bard & Faulkner, *Electrochemical Methods*, 2nd ed.). At pH 0, $E^\circ_{\mathrm{Cu^{2+}/Cu}} = +0.34\mathrm{V}$ and $E^\circ_{\mathrm{2H^+/H_2}} = 0.00\mathrm{V}$

vs. SHE, with pH-dependent shifts applied via Equation S29. Standard potentials and Gibbs free energies for NO reduction to N_2O, N_2, and NH_3 were obtained from thermodynamic compilations (e.g., NIST Chemistry WebBook and other standard thermodynamic data sets) and converted to effective one-step standard potentials for Equations S25-S27. These values were then adjusted for the working pH using Equation S29. Because the main objective was to bound the FE of NO reduction rather than to resolve detailed mechanistic subtleties, the NO pathways were treated as lumped overall reactions with effective standard potentials and kinetic prefactors.

For Cu deposition and the HER, representative $j_{0,i}$ values are taken directly from experimental studies. For the three NO-reduction pathways, explicit j_0 values for bare (or near-bare) Cu in aqueous solution are not available; instead, we selected conservative upper bounds based on reported NO electroreduction current densities and Tafel slopes, and treat them as model parameters constrained by literature, rather than strict experimental constants.

The total local current density is given by the sum over all pathways:

$$j_{\mathrm{tot}}(E) \;=\; \sum_i j_i\,(E) \tag{S30}$$

and the applied current density j_{app}(A m^{-2}) is imposed through the condition:

$$j_{\mathrm{tot}}(E^*) \;=\; j_{\mathrm{app}} \tag{S31}$$

which implicitly defines the operating potential E^* for a given set of bulk concentrations and kinetic parameters. At this operating point, the Faradaic efficiency of reaction i is:

$$\mathrm{FE}_i \;=\; \frac{j_i(E^*)}{j_{\mathrm{tot}}(E^*)} \;=\; \frac{j_i(E^*)}{j_{\mathrm{app}}} \tag{S32}$$

Mass Transport and NO Availability at the Cathode

Because NO enters the cell from the gas side and must dissolve and diffuse through the electrolyte to reach the reactive sites in the GDE, its surface concentration was limited by Henry's law and diffusion through a stagnant diffusion layer. The equilibrium aqueous concentration of NO at the gas-liquid interface, C^*_{NO} (mol m^{-3}), was obtained from Henry's law:

$$C^*_{\mathrm{NO}} \;=\; k_{H,\mathrm{NO}}\, P_{\mathrm{NO}} \tag{S33}$$

where P_{NO} is the NO partial pressure (Pa) and $k_{H,\mathrm{NO}}$ is the Henry coefficient (mol m^{-3} Pa^{-1}). The Henry constant was taken from standard compilations of gas solubility and diffusion data; for nitric oxide at 25 °C, $H'_{\mathrm{NO}} \approx 2.9 \times 10^9$ Pa, corresponding to a diffusion coefficient in water of $D_{\mathrm{NO}} \approx$

$2.4 \times 10^{-9}\,\mathrm{m^2\,s^{-1}}$. These values are consistent with typical transport parameters used for dissolved small molecules in aqueous electrolytes.

A one-dimensional stagnant-film approximation was used to estimate the maximum diffusion-limited flux of NO toward the cathode surface:

$$j_{\text{lim,NO}} = n_{\mathrm{NO}}\, F\, \frac{D_{\mathrm{NO}}}{\delta}\, C^{*}_{\mathrm{NO}} \tag{S34}$$

where δ is an effective diffusion layer thickness (m) and n_{NO} is the electron stoichiometry of the dominant NO reduction pathway (taken as 4 for estimating $j_{\text{lim,NO}}$). For gas-diffusion electrodes operating under forced convection or gas-channel flow, δ is typically on the order of 10-50 μm. In the model, δ was taken as 50 μm to provide a conservative upper bound on NO availability at the surface.

The kinetic current density predicted by Equation S28 for each NO pathway, $j^{\text{kin}}_{\mathrm{NO},i}$, was then compared to the diffusion-limited value; the actual NO-related current was taken as:

$$j_{\mathrm{NO},i} = \min\left(j^{\text{kin}}_{\mathrm{NO},i},\ f_i\, j_{\text{lim,NO}}\right) \tag{S35}$$

where f_i is the fractional distribution to each NO product pathway (with $\sum_i f_i = 1$). This construction guarantees that the sum of all NO-related currents cannot exceed the diffusion-limited supply of NO to the surface.

Simulation Conditions and Parameter Ranges

Simulations were performed for NO concentrations in the range 200-1000 ppm (i.e., $P_{\mathrm{NO}} = 2 \times 10^{-4}$ to 1×10^{-3} bar), spanning typical values reported for both natural gas and coal flue gases. The pH and bulk $Cu(EDA)_2^{2+}$ concentrations were set to match the experimentally relevant values for the optimized operating conditions. For each combination of P_{NO}, pH, and j_{app}, Equations S28-S31 were solved to obtain E^*, the partial current densities $j_i(E^*)$, and the corresponding Faradaic efficiencies via Equation S32.

S6. SMEC PACKAGING DESIGN & POWER-PLANT INTEGRATION ANALYSIS

A detailed system-level framework was developed to translate single-cell electrochemical performance into container-scale module layouts and ultimately into fleet-level deployment requirements for power-plant decarbonization. First, the SMEC packaging is described, then the number of SMEC units required for decarbonizing a 360 MW natural gas plant was determined.

S6.1. SMEC Packaging and System Configuration

A detailed packaging and spatial-allocation model was constructed for a 40-foot ISO shipping container (internal dimensions 12.03 m × 2.35 m × 2.39 m). The objective of this analysis was to determine the maximum electrochemically active surface that can be housed within a standard container while preserving adequate space for headers, manifolding, power distribution hardware, thermal management components, and maintenance access. All dimensions, parameters, and calculated values reported here are sufficient to allow exact reconstruction of the SMEC layout.

The design begins at the individual cell level. Each electrochemical cell consists of a 20 cm × 20 cm active area (400 cm^2), consistent with the 300-500 cm^2 electrode footprints typically used in stationary proton exchange membrane (PEM) fuel cells and electrolyzers, where manufacturability, uniform gas distribution, and thermal control impose similar constraints. Each cell requires approximately 11 mm of thickness for the electrode/electrolyte assembly (10 mm for the electrolyte and 2 × 0.5 mm GDEs) and an additional ~1 mm for the bipolar plate, for a nominal 12 mm cell pitch. Seventy-five such cells, together with 60 mm end-plate hardware (2 × 30 mm) and 2 mm collector plates, yield a stack thickness of 0.962 m. Within the container cross-section, the stacks are arranged in a 10 × 10 grid, occupying 2.00 m × 2.00 m. This layout leaves 0.35 m (width) and 0.39 m (height) of free space to route gas manifolds, cooling lines, electrical buses, and monitoring hardware. Nine identical modules are placed longitudinally along the 12.03 m interior length. The total occupied length is the sum of nine stack depths, eight inter-module gaps (each 60 mm), and 200 mm of end clearance on both ends: $L_{occ} = 9(0.962m) + 8(0.06m) + 2(0.20m) = 9.538m$. The fraction of the container length utilized is therefore: $\phi = 9.538\ m / 12.03\ m = 0.793$. This indicates that approximately 80% of the container interior is devoted to electrochemical hardware. This 80% fill ratio lies within the range typically adopted for containerized process skids, where 15-25% unused length is necessary to house power electronics, ventilation manifolds, fluid distribution headers, and to provide essential maintenance access.

Each SMEC container therefore houses nine modules, each composed of a 10 × 10 matrix of 75-cell stacks, for a total of 67,500 electrochemical cells. Every cell exposes a 400 cm^2 geometric GDE surface to the flue-gas stream. The porous carbon GDE provides approximately an eightfold enhancement in effective electrochemical surface area due to its microporous architecture and internal pore network, representing an electrochemical active surface area to geometric area ratio (ECAS-to-GA) of 8. Thus, each cell provides an active surface: A_{active} = 8 × 400 cm^2 = 3,200 cm^2 = 0.32 m^2, and the total active area per container is: A_{total} = 67,500 × 0.32 m^2 = 21,600 m^2. This extremely large aggregate interfacial area is the primary enabler of high mass-transfer rates and rapid CO_2 turnover required for megawatt-scale operation. These packaging assumptions provide a reproducible baseline for conducting sensitivity analyses on current density, ECAS-to-GA ratio, and other performance parameters. Minor variations in porosity factor, cell count, or spacing can be incorporated into the model without significant deviations from the overall findings, due to the substantial active-area margin incorporated in the baseline design. The following table summarizes all the SMEC packaging and configurations.

Table S3. SMEC packaging dimensions and stack configuration for a 40-ft ISO container

Parameter	Symbol	Value	Units	Physical Meaning	Justification / Source of Assumption
Container internal length	L_n	12.03	m	Usable longitudinal space in an ISO container	Standard 40-ft ISO Type IIC specification
Container internal width	W_n	2.35	m	Usable width for stack cross-section + clearances	ISO Type IIC specification
Container internal height	H_n	2.39	m	Usable internal height for stack + overhead routing	ISO Type IIC specification
Cell geometric dimensions	—	0.20 × 0.20	m	Lateral dimensions of each GDE face	Matches commercial PEMFC/PEM electrolysis cell format; supports uniform flow distribution
Cell geometric area	A_{cell}	0.04	m^2	Planform area available for gas contact	= 0.20 m × 0.20 m
Electrochemical active surface area multiplier	ECAS-to-GA	8	–	Ratio of true active area to geometric area due to porosity	Consistent with carbon-based GDL+microporous layer porosity and BET surface area in commercial GDEs

Active area per cell	A_{active}	0.32	m^2	Effective CO_2 absorption area	A_{cell} × ECAS-to-GA
Cells per stack	N_{cell}	75	—	Number of repeating unit cells in each bipolar stack	Consistent with stationary PEM stacks (50-100 cells)
Electrode + separator + frame thickness per cell	d_{cell}	11	mm	Includes cathode GDE, anode GDE, electrolyte spacer/gasket	Consistent with PEM cell hardware (10-14 mm)
Bipolar plate (BPP) thickness	d_{BPP}	1	mm	Conductive separator plate	Thin BPPs common in polymer-electrolyte stacks
Endplate thickness	$d_{endplate}$	30 per plate	mm	Structural compression plate	Needed for uniform clamping pressure (60 mm per stack)
Current collector thickness	$d_{collector}$	1	mm	Conductive bus connection	Standard for stationary stacks
Total stack thickness	d_{stack}	0.962	m	Total depth of a 75-cell stack	Computed: 75 × (0.011 + 0.001) + 0.060 + 0.002
Module cross-section	L_{cross}, W_{cross}	2.00 × 2.00	m	10 × 10 cell array	= 10 cells × 0.20 m dimension
Side clearance (width)	—	0.35	m	Clearance for manifolds, cabling	$W_n - W_{cross} = 2.35 - 2.00 = 0.35$ m
Top clearance (height)	—	0.39	m	Cooling, wiring, and service gap	$H_n - L_{cross} = 2.39 - 2.00 = 0.39$ m
Modules per SMEC container	N_{mod}	9	—	Number of electrochemical modules arranged along the length	Optimized to maximize packing while preserving service access
Inter-module gap	Δ_{gap}	0.06	m	Spacing between modules	Allows headers, busbars, and mechanical tolerances
End clearance	Δ_{end}	0.2	m	Clearance at each end for bulkheads	Needed for electrical and fluid interconnects
Occupied container length	L_{occ}	9.538	m	Total module + gap length	$9d_{stack} + 8\Delta_{gap} + 2\Delta_{end}$
Fraction of container filled	ϕ	0.793	—	Space utilization ratio	$L_{occ} / L_n = 9.538/12.03 = 0.793$
Total number of cells per container	$N_{cells,SMEC}$	67,500	—	Number of electrochemical cells in one SMEC	75 × (10 × 10) × 9
Total active area in the container	A_{total}	21,600	m^2	Effective mass-transfer area	$A_{active} \times N_{cells,SMEC}$

S6.2. Power-Plant Case Study and SMEC Fleet Sizing Framework

In this section, we elaborated on the number of SMEC units needed for decarbonization of the power plant of the data center. The turbine operates at a nominal capacity of 360 MW and a capacity factor of 0.60, corresponding to typical baseload operation for gas turbines paired with renewable integration. The CO_2 emission factor was taken as 0.4 tonnes CO_2 per MWh of electricity generated, a widely used value for modern natural-gas combined-cycle systems. Combining these inputs yields an annual CO_2 emission rate of 7.57×10^5 tonnes. This value represents the total annual CO_2 load that the SMEC fleet must treat under the various decarbonization scenarios examined.

The full parameter set for the power plant is summarized in the following table:

Table S4. Power plant operating parameters and annual CO_2 emissions

Parameter	Symbol	Value	Units	Physical Meaning	Source/ Justification
Plant electrical capacity	P_{plant}	360	MW	Rated output of the gas turbine	Case-study specification
Capacity factor	CF	0.6	–	Fraction of the year the plant operates at load	Typical NGCC CF for baseload service
Annual operating hours	–	8,760	h/yr	Full year	Calendar
CO_2 emission factor	EF	0.40	tonneCO_2/MWh	Emissions per electrical output	Standard NGCC values
Annual CO_2 emissions	E_{tot}	7.57×10^5	tonneCO_2/yr	Total emissions to be captured	Calculated from above

Per-SMEC CO_2 Capture Capacity

The capture capacity of one SMEC container depends on the total electrochemically active surface area housed within the 40-ft container (Section S5.1), the applied current density, the annual operating time, and Faraday's law. The active area per container (A_{active}) established previously is 21,600 m^2. For a given applied current density j (A m^{-2}), the total annual charge

passed through a single SMEC is: Q = A_{active} × j × t_{op}, where the annual operating time (t_{op}) is: t_{op} = 365 × 24 × 3,600 = 1.892 × 10^7 s. Using Faraday's law, the annual moles of electrons delivered are (Q/F). For the EMAR cell, one electron corresponds to one CO_2 molecule released from carbamate during anodic regeneration (i.e., n_e=1). Thus, the annual mass of CO_2 captured by one SMEC is: C_{SMEC} = Q/F × 44 g mol^{-1}.

Two performance configurations, consistent with the main text, are analyzed:

- *Current-state*: ECAS-to-GA = 8, j = 200 A m^{-2}
- *Future-development*: ECAS-to-GA = 12, j = 500 A m^{-2}

These values reflect realistic progress in GDE microstructure, catalyst distribution, and mass-transport optimization. ECAS-to-GA enhancements above 10× are already reported for nanostructured carbon and metal-foam architectures [35, 36], while current densities of 500-1000 A m^{-2} are common in stationary PEM electrolyzers and high-utilization fuel-cell stacks [37, 38]. The full set of electrochemical parameters is summarized in the following table:

Table S5. Electrochemical performance parameters and operating assumptions for the estimation of CO_2 capture capacity per SMEC container

Parameter	Symbol	Value(s)	Units	Notes / Physical Meaning
Active area per SMEC	A_{active}	21,600	m^2	From Section S5.1 packaging
Annual operating time	t_{op}	1.892 × 10^7	s	Continuous operation assumption
Faraday constant	F	96,485	C mol^{-1}	–
Electron stoichiometry	n_e	1	–	One e^- regenerates one CO_2
Current density (current-state)	j	200	A m^{-2}	Conservative PEM-derived
Current density (future-state)	j	500	A m^{-2}	Projected with next-gen GDE
ECAS-to-GA (current/future)	α	8/12	–	Matches main-text performance tiers

Using these parameters, the annual CO_2 capture capacity per SMEC for each performance tier is: $C_{SMEC} = \frac{A_{active}\, j\, t_{op}}{F} \times 44$. The results for the number of SMEC required for each scenario are provided in the following table:

Table S6. Number of SMECs required for three CO_2 capture capacity scenarios

Scenario	Removal Fraction, *R*	*Current-state*	*Future-development*
Ideal **decarbonization**	0.98	19.9	5.3
Typical **decarbonization**	0.90	18.3	4.9
Partial **decarbonization**	0.50	10.2	2.7

Interpretation and Justification

The results demonstrate that even in the conservative, current-state configuration, decarbonization of a 360 MW natural-gas power plant requires fewer than twenty SMEC containers for near-complete (90-98%) CO_2 removal. This remarkably small physical footprint arises directly from the 21,600 m^2 of active surface area that can be packaged into a single 40-ft container. In the future development scenarios, the required SMEC count drops to around five for near-complete decarbonization.

The parameters used are technically justified. The ECAS-to-GA values (8 and 12) correspond to electrochemically active surface area multipliers routinely observed in carbon-paper GDEs (5-8×), carbon foams (10-15×), and nanostructured metal foams (>12×) [35, 36, 39]. The current densities (200 and 500 A m^{-2}) are reasonable relative to commercial PEM electrolyzers. In combination, these results illustrate that containerized SMEC technology can deliver multi-MW carbon capture with minimal spatial footprint, tunable performance via electrode design, and predictable scaling relationships suitable for deployment in energy-intensive AI data-center infrastructures.

S7. TECHNO-ECONOMIC ANALYSIS

A techno-economic analysis (TEA) was conducted to assess the economic impact of carbon capture on the levelized cost of electricity (LCOE) for OpenAI's Stargate natural gas power plant retrofitted with the carbon-capture modules. Using the U.S. Department of Energy LCOE calculator, the reference facility located in Abilene, Texas, was estimated to have a baseline LCOE of 58.4 $/MWh. Table S7 summarizes the principal input assumptions adopted in the LCOE analysis. These assumptions are consistent with those commonly applied in TEAs of comparable electrochemical energy technologies [8, 35, 40, 41].

Table S7. General assumptions for LCOE calculation

Economic Parameter	Value	Unit
Plant lifetime	25	years
Interest rate	10	%
Capital recovery factor	11	%
Electricity price	58.4	\$/MWh
GDE Price	15	$\$/m^2$
Gasket/Sealing Price	200	$\$/m^2$
Bipolar Plate Price	200	$\$/m^2$
Current Collector Price	3000	$\$/m^2$
Stack Assembly	10	% of stack components
Manifold and Other Module Assembly	10	% of module components
Container Price	5000	$

Capital expenses (CapEx) and operating expenses (OpEx) were evaluated to estimate the resulting change in LCOE. Within the CapEx breakdown, costs were governed primarily by the purchased equipment cost (PEC), which was divided into (*i*) conventional process equipment (e.g., compressor) and (ii) electrochemical components. The process-related PEC was derived from equipment sizing and published data for an EDA-based carbon-capture unit [8]. In contrast, the electrochemical PEC was calculated using the assumptions in Table S5, along with the sizing and discussion presented in Section S5.

Total CapEx was annualized through depreciation by applying the capital recovery factor (*CRF*) to the fixed capital investment (*FCI*). The *CRF* was calculated from the interest rate (*i*) and plant lifetime (*n*) according to Eq. S37, while the *FCI* was estimated via the percentage of delivered equipment cost method (Eq. S38) [42].

$$CapEx_{with\ depreciation} = CRF \times FCI \quad \text{(S36)}$$

$$CRF = \frac{i*(1+i)^n}{(1+i)^n-1} \quad \text{(S37)}$$

$$FCI = \sum FCI_i \quad \text{(S38)}$$

$$FCI_i = C_i \sum(1 + f_1 + f_2 + \dots + f_n) \quad \text{(S39)}$$

where the factors f_1, f_2, f_3, …, f_n denote cost factors accounting for additional direct and indirect items (e.g., piping, electrical and instrumentation systems, and other overheads). The values of these factors are reported in Table S8, adapted from Ref. [42]. The term C_i corresponds to the purchase cost of equipment item *i*:

$$C_i = C_{i,ref} \times \left(\frac{A_i}{A_{i,ref}}\right)^k \times \left(\frac{CEPCI_{2023}}{CEPCI_{ref}}\right) \quad \text{(S40)}$$

where $C_{i,ref}$ represents the reference equipment cost, $A_{i,ref}$ is the reference equipment size, and A_i is the equipment size in this study. *k* is the scaling factor, which is assumed to be 0.6 [43]. Additionally, the equipment costs were updated to recent values by the Chemical Engineering Plant Cost Index (*CEPCI*).

Table S8. Ratio factors for estimating capital investment items based on Delivered-Equipment Cost. The ratios used are suitable for a solid-fluid processing plant and are adapted from ref. [42].

**Direct Costs**	**Fraction of delivered equipment cost (f_i)**
Purchased equipment installation	0.39
Instrumentation & controls (installed)	0.26
Piping (installed)	0.31
Electrical systems (installed)	0.10
Buildings (including services)	0.29
Yard improvements	0.12
Service facilities (installed)	0.55

Total Direct Costs	*2.02*
**Indirect Costs**	
Engineering and supervision	0.32
Construction expenses	0.34
Legal expenses	0.04
Contractor's fee	0.19
Contingency	0.37
Total Indirect Costs	*1.26*

Table S9. The purchased costs for the SMEC process

Equipment	Size, A_i	Purchased Equipment Cost, C_i (M$)
**Inlet gas blower**	0.55 [a]	2.53
**Boiler**	2.46×10^5 [b]	3.71
**Condenser**	13.5 [c]	0.73
**Compressor**	9650 [d]	7.59
**SMEC** [e]	–	17.82
**Total**	–	32.4

[a] Flowrate in MMkg/h
[b] Steam flowrate in m^3/h
[c] Condenser duty in MWe
[d] Discharge pressure in kPa
[e] Based on calculations in Section S6.2

OpEx consists of two feedstock-related and non-feedstock cost categories. Feedstock-related OpEx mainly includes the electricity consumption necessary for system operation, whereas non-feedstock OpEx comprises personnel costs, on-site feedstock handling, and routine maintenance activities. These two categories were estimated using the following equations:

$$OpEx = OpEx_{feedstock} + OpEx_{non-feedstock} \tag{S41}$$

$$OpEx_{feedstock} = I\ U\ N_{hr}\ C_{electrity} \tag{S42}$$

where N_{hr} denotes the annual operating hours corresponding to a 60% capacity factor, and $C_{electricity}$ represents the reference electricity price, taken as 58.4 $/MWh. Non-feedstock OpEx is approximated as 5% of the annualized CapEx, consistent with recommendations in prior TEA studies [44-46].

$$OpEx_{non-feedstock} = 0.05 \times CapEx_{with\ depreciation} \quad \text{(S43)}$$

Finally, LCOE was calculated by considering both annual CapEx and OpEx and normalizing by the powerplant capacity (MWh), which is the product of power and annual operation hours:

$$LCOE = 58.4 + \frac{CapEx_{with\ depreciation} + OpEx}{P_{plant} \times CF \times 8760} \quad \text{(S44)}$$

Benchmark CO_2 capture cost values for established carbon capture technologies were compiled from multiple literature sources, including reports from the International Energy Agency, the Global CCS Institute, and Lawrence Livermore National Laboratory [1–5]. Cost ranges reported for different types of power plants, including coal-fired power generation, natural gas-fired power generation, and waste biomass facilities, were used as reference cases for comparison. These reported values span a broad range because capture costs are strongly influenced by the selected separation technology, scale and CO_2 emission rate, degree of process integration, and location-specific economic assumptions. For instance, reported capture costs for coal-fired power plants vary from $20 to $132 per tonne of CO_2, while those for natural gas-fired power plants generally fall between $49 and $150 per tonne of CO_2. Because the referenced studies express the levelized cost of carbon capture (LCOCC) in $ per tonne of CO_2, the TEA results for the SMEC process are reported using the same basis to enable direct comparison. TEA of the SMEC system indicates that, under the representative capture case, the LCOCC decreases from $39.7/tonne CO_2 for the current system configuration to $32.3/tonne CO_2 in the future development scenario. Under the partial decarbonization case, the corresponding LCOCC values are $42.8 and $35.4 per tonne of CO_2 for the current and future configurations, respectively. These results demonstrate the potential economic competitiveness of SMEC compared to the conventional processes due to the avoidance of using thermal energy and process simplification through using modular membrane-less SMEC containers.

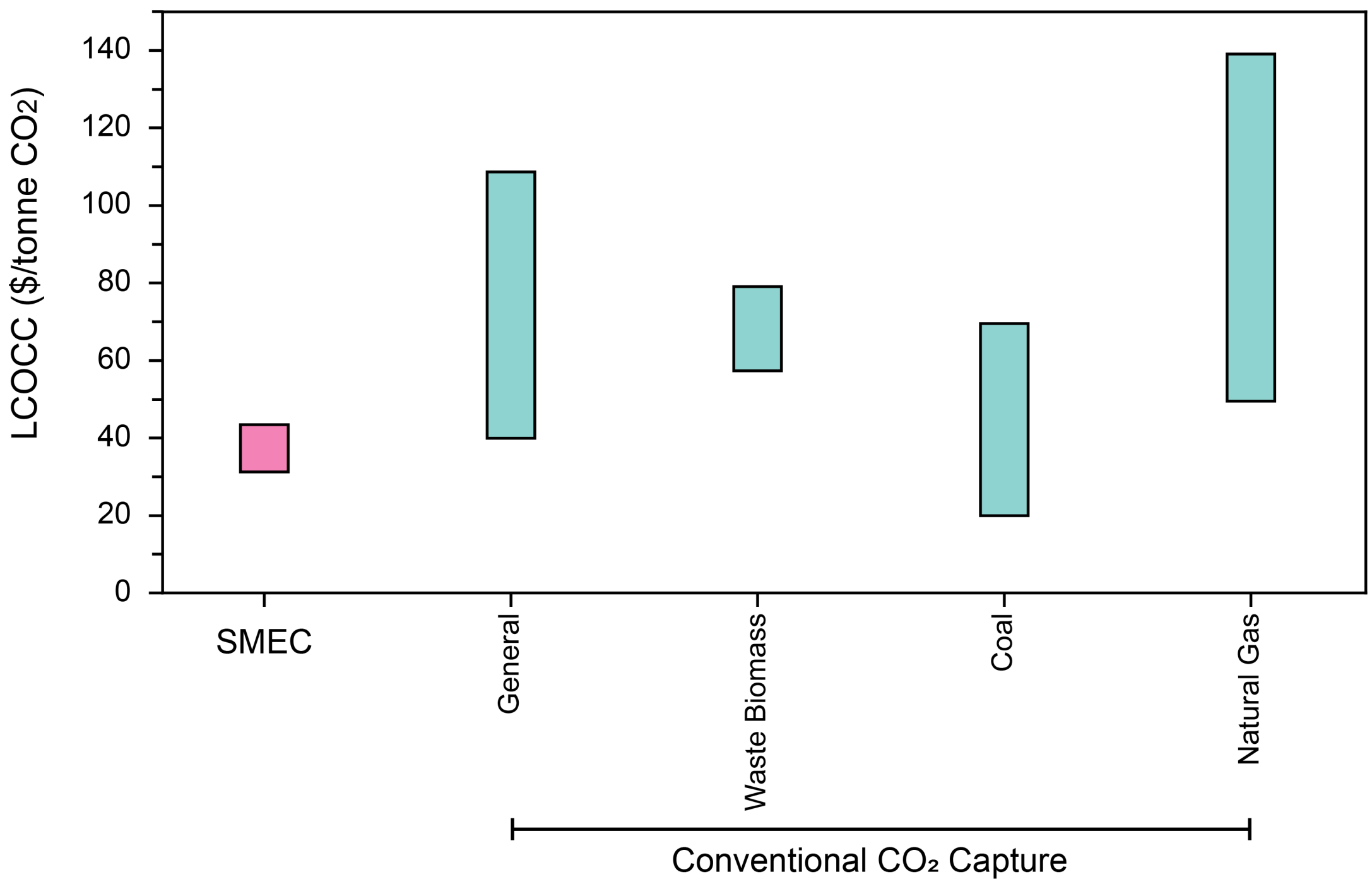

Figure S8. A comparison between the economics of SMEC and conventional CO_2 capture from power plants.

S8. SUPPLEMENTARY REFERENCES

1. Stern, M.C., F. Simeon, H. Herzog, and T.A. Hatton, *Post-combustion carbon dioxide capture using electrochemically mediated amine regeneration.* Energy & Environmental Science, 2013. **6**(8): p. 2505-2517.

2. Stern, M.C. and T.A. Hatton, *Bench-scale demonstration of CO 2 capture with electrochemically-mediated amine regeneration.* RSC Advances, 2014. **4**(12): p. 5906-5914.

3. Stern, M.C., *Electrochemically-mediated amine regeneration for carbon dioxide separations*. 2013, Massachusetts Institute of Technology.

4. Wang, M., H.J. Herzog, and T.A. Hatton, *CO2 capture using electrochemically mediated amine regeneration.* Industrial & Engineering Chemistry Research, 2020. **59**(15): p. 7087-7096.

5. Rahimi, M., K.M. Diederichsen, N. Ozbek, M. Wang, W. Choi, and T.A. Hatton, *An electrochemically mediated amine regeneration process with a mixed absorbent for postcombustion CO2 capture.* Environmental Science & Technology, 2020. **54**(14): p. 8999-9007.

6. Wang, M., M. Rahimi, A. Kumar, S. Hariharan, W. Choi, and T.A. Hatton, *Flue gas CO2 capture via electrochemically mediated amine regeneration: System design and performance.* Applied Energy, 2019. **255**: p. 113879.

7. Wang, M., S. Hariharan, R.A. Shaw, and T.A. Hatton, *Energetics of electrochemically mediated amine regeneration process for flue gas CO2 capture.* International Journal of Greenhouse Gas Control, 2019. **82**: p. 48-58.

8. Wang, M., R. Shaw, E. Gencer, and T.A. Hatton, *Technoeconomic analysis of the electrochemically mediated amine regeneration CO2 capture process.* Industrial & Engineering Chemistry Research, 2020. **59**(31): p. 14085-14095.

9. Rahimi, M., F. Zucchelli, M. Puccini, and T. Alan Hatton, *Improved CO2 capture performance of electrochemically mediated amine regeneration processes with ionic surfactant additives.* ACS Applied Energy Materials, 2020. **3**(11): p. 10823-10830.

10. Fan, H., Y. Mao, S. Sultan, Y. Yu, X. Wu, and Z. Zhang, *Performance enhancement of desorption reactor in the electrochemically mediated amine regeneration CO2 capture process: Thru modelling, simulation, and optimization.* Applied Energy, 2024. **376**: p. 124287.

11. Wu, X., H. Fan, M. Sharif, Y. Yu, K. Wei, Z. Zhang, and G. Liu, *Electrochemically-mediated amine regeneration of CO2 capture: From electrochemical mechanism to bench-scale visualization study.* Applied Energy, 2021. **302**: p. 117554.

12. Hassan, A., A. Refaie, P. Aleta, M. Afshari, E. Kalantari, Y. Fang, and M.M. Rahimi, *Reviving the absorbent chemistry of electrochemically mediated amine regeneration for improved point source carbon capture.* Chemical Engineering Journal, 2024: p. 149566.

13. Fan, H., Y. Mao, H. Wang, Y. Yu, X. Wu, and Z. Zhang, *Performance comparation of MEA and EDA in electrochemically-mediated amine regeneration for CO2 capture.* Separation and Purification Technology, 2023. **311**: p. 123282.

14. Wu, X., Y. Mao, H. Fan, S. Sultan, Y. Yu, and Z. Zhang, *Investigation on the performance of EDA-based blended solvents for electrochemically mediated CO2 capture.* Applied Energy, 2023. **349**: p. 121656.

15. Liang, L., F. Firschke, J. Wang, L. Yang, X. Wang, W. Ju, M.T. Mayer, and P. Strasser, *Anion effects govern efficiency of electrochemical amine-mediated CO2 capture/release.* Nature Communications, 2025.

16. Hassan, A., M. Afshari, and M. Rahimi, *A membraneless electrochemically mediated amine regeneration for carbon capture.* Nature Communications, 2025. **16**(1): p. 6333.

17. Mao, Y., H. Fan, Y. Yang, J. Liu, Y. Yu, Z. Zhang, and X. Wu, *Mitigating cathode blocking in the advanced electrochemically mediated amine regeneration CO_2 capture system by asymmetric copper foam electrode design.* Separation and Purification Technology, 2026. **382**: p. 135825.

18. Ali, M., R. Saidur, and M. Hossain, *A review on emission analysis in cement industries.* Renewable and Sustainable Energy Reviews, 2011. **15**(5): p. 2252-2261.

19. Mahasenan, N., S. Smith, and K. Humphreys. *The cement industry and global climate change: current and potential future cement industry CO2 emissions*. in *Greenhouse gas control technologies-6th international conference*. 2003. Elsevier.

20. Kim, Y. and E. Worrell, *International comparison of CO2 emission trends in the iron and steel industry.* Energy policy, 2002. **30**(10): p. 827-838.

21. Gouedard, C., D. Picq, F. Launay, and P.-L. Carrette, *Amine degradation in CO2 capture. I. A review.* International journal of greenhouse gas control, 2012. **10**: p. 244-270.

22. Rochelle, G.T., *Thermal degradation of amines for CO2 capture.* Current Opinion in Chemical Engineering, 2012. **1**(2): p. 183-190.

23. Gao, H., Z. Liang, H. Liao, and R.O. Idem, *Thermal degradation of aqueous DEEA solution at stripper conditions for post-combustion CO2 capture.* Chemical engineering science, 2015. **135**: p. 330-342.

24. Zhai, Y., S. Gong, W. Li, Q. Xia, T. Li, J. Guan, S.-Y. Leu, Z.-Y. Wu, S.P. Lau, and X. Zhang, *Long-term electrochemical carbon capture from diverse CO2 sources with a recirculation mode.* Nature Communications, 2025. **16**(1): p. 10389.

25. Liu, T., Y. Wang, Y. Wu, W. Jiang, Y. Deng, Q. Li, C. Lan, Z. Zhao, L. Zhu, and D. Yang, *Continuous decoupled redox electrochemical CO2 capture.* Nature Communications, 2024. **15**(1): p. 10920.

26. Jin, X., S. Jin, L. Li, R.G. Gordon, P. Wang, M.J. Aziz, and Y. Ji, *Direct air capture of CO2 in an electrochemical hybrid flow cell with a spatially isolated phenazine electrode.* Nature Energy, 2025. **10**(9): p. 1146-1154.

27. Zhu, P., Z.-Y. Wu, A. Elgazzar, C. Dong, T.-U. Wi, F.-Y. Chen, Y. Xia, Y. Feng, M. Shakouri, and J.Y. Kim, *Continuous carbon capture in an electrochemical solid-electrolyte reactor.* Nature, 2023. **618**(7967): p. 959-966.

28. Jin, S., M. Wu, Y. Jing, R.G. Gordon, and M.J. Aziz, *Low energy carbon capture via electrochemically induced pH swing with electrochemical rebalancing.* Nature Communications, 2022. **13**(1): p. 2140.

29. Pang, S., S. Jin, F. Yang, M. Alberts, L. Li, D. Xi, R.G. Gordon, P. Wang, M.J. Aziz, and Y. Ji, *A phenazine-based high-capacity and high-stability electrochemical CO2 capture cell with coupled electricity storage.* Nature Energy, 2023. **8**(10): p. 1126-1136.

30. Li, X., X. Zhao, Y. Liu, T.A. Hatton, and Y. Liu, *Redox-tunable Lewis bases for electrochemical carbon dioxide capture.* Nature Energy, 2022. **7**(11): p. 1065-1075.

31. Abdinejad, M., M. Massen-Hane, H. Seo, and T.A. Hatton, *Oxygen-Stable Electrochemical CO2 Capture using Redox-Active Heterocyclic Benzodithiophene Quinone.* Angewandte Chemie International Edition, 2024. **63**(52): p. e202412229.

32. Liu, Y., H.-Z. Ye, K.M. Diederichsen, T. Van Voorhis, and T.A. Hatton, *Electrochemically mediated carbon dioxide separation with quinone chemistry in salt-concentrated aqueous media.* Nature communications, 2020. **11**(1): p. 2278.

33. Zhang, X., Z. Fang, P. Zhu, Y. Xia, and H. Wang, *Electrochemical regeneration of high-purity CO2 from (bi) carbonates in a porous solid electrolyte reactor for efficient carbon capture.* Nature Energy, 2025. **10**(1): p. 55-65.

34. Bui, J.C., É. Lucas, E.W. Lees, A.K. Liu, H.A. Atwater, C. Xiang, A.T. Bell, and A.Z. Weber, *Analysis of bipolar membranes for electrochemical CO 2 capture from air and oceanwater.* Energy & Environmental Science, 2023. **16**(11): p. 5076-5095.

35. Minke, C., U. Kunz, and T. Turek, *Carbon felt and carbon fiber-A techno-economic assessment of felt electrodes for redox flow battery applications.* Journal of Power Sources, 2017. **342**: p. 116-124.

36. Liu, Z., X. Yuan, S. Zhang, J. Wang, Q. Huang, N. Yu, Y. Zhu, L. Fu, F. Wang, and Y. Chen, *Three-dimensional ordered porous electrode materials for electrochemical energy storage.* NPG Asia Materials, 2019. **11**(1): p. 12.

37. Sharaf, O.Z. and M.F. Orhan, *An overview of fuel cell technology: Fundamentals and applications.* Renewable and Sustainable Energy Reviews, 2014. **32**: p. 810-853.

38. David, M., C. Ocampo-Martínez, and R. Sánchez-Peña, *Advances in alkaline water electrolyzers: A review.* Journal of Energy Storage, 2019. **23**: p. 392-403.

39. Kim, J., D. Lee, C. Kim, H. Lee, S. Baek, and J.H. Moon, *Electrochemically active porous carbon nanospheres prepared by inhibition of pyrolytic condensation of polymers.* Proceedings of the National Academy of Sciences, 2023. **120**(19): p. e2222050120.

40. Prykhodko, Y., K. Fatyeyeva, L. Hespel, and S. Marais, *Progress in hybrid composite Nafion®-based membranes for proton exchange fuel cell application.* Chemical Engineering Journal, 2021. **409**: p. 127329.

41. James, B.D., J.M. Huya-Kouadio, C. Houchins, and D.A. DeSantis, *Mass Production Cost Estimation of Direct H2 PEM Fuel Cell Systems for Transportation Applications: 2018 Update.* report by Strategic Analysis, Inc., under Award Number DE-EE0007600 for the US Department of Energy, 2018.

42. Peters, M.S., K.D. Timmerhaus, and R.E. West, *Plant design and economics for chemical engineers*. Vol. 4. 2003: McGraw-hill New York.

43. *Economic Indicators.* Chemical Engineering, 2024. **122**(2): p. 48-48.

44. Alerte, T., A. Gaona, J.P. Edwards, C.M. Gabardo, C.P. O'Brien, J. Wicks, L. Bonnenfant, A.S. Rasouli, D. Young, J. Abed, L. Kershaw, Y.C. Xiao, A. Sarkar, S.A. Jaffer, M.W. Schreiber, D. Sinton, H.L. MacLean, and E.H. Sargent, *Scale-Dependent Techno-Economic Analysis of CO2 Capture and Electroreduction to Ethylene.* ACS Sustainable Chemistry & Engineering, 2023. **11**(43): p. 15651-15662.

45. Arndt, B.M., B. Willson, and M. Nazemi, *Thermodynamic and Economic Assessments of Electrochemical CO2 Conversion to Dimethyl Ether: Trade-off between Hydrogen Gas and Water Vapor as a Proton Source.* Industrial & Engineering Chemistry Research, 2024. **63**(33): p. 14582-14589.

46. Compagnoni, M., E. Mostafavi, A. Tripodi, N. Mahinpey, and I. Rossetti, *Techno-economic Analysis of a Bioethanol to Hydrogen Centralized Plant.* Energy & Fuels, 2017. **31**(11): p. 12988-12996.
